# Wall mechanics and exocytosis define the shape of growth domains in fission yeast

**DOI:** 10.1038/ncomms9400

**Published:** 2015-10-12

**Authors:** Juan F. Abenza, Etienne Couturier, James Dodgson, Johanna Dickmann, Anatole Chessel, Jacques Dumais, Rafael E. Carazo Salas

**Affiliations:** 1 Genetics Department, University of Cambridge, Downing Street, Cambridge, CB2 3EH, UK; 2 Wellcome Trust/Cancer Research UK Gurdon Institute, University of Cambridge, Tennis Court Road, Cambridge, CB2 1QN, UK; 3 Departamento de Física, Universidad de Santiago de Chile, Santiago, Chile; 4 Facultad de Ingeniería y Ciencias, Universidad Adolfo Ibáñez, Viña del Mar 2562307, Chile; 5 Department of Organismic and Evolutionary Biology, Harvard University, 16 Divinity Avenue, Cambridge, Massachusetts 02138, USA

## Abstract

The amazing structural variety of cells is matched only by their functional diversity, and reflects the complex interplay between biochemical and mechanical regulation. How both regulatory layers generate specifically shaped cellular domains is not fully understood. Here, we report how cell growth domains are shaped in fission yeast. Based on quantitative analysis of cell wall expansion and elasticity, we develop a model for how mechanics and cell wall assembly interact and use it to look for factors underpinning growth domain morphogenesis. Surprisingly, we find that neither the global cell shape regulators Cdc42-Scd1-Scd2 nor the major cell wall synthesis regulators Bgs1-Bgs4-Rgf1 are reliable predictors of growth domain geometry. Instead, their geometry can be defined by cell wall mechanics and the cortical localization pattern of the exocytic factors Sec6-Syb1-Exo70. Forceful re-directioning of exocytic vesicle fusion to broader cortical areas induces proportional shape changes to growth domains, demonstrating that both features are causally linked.

## Abstract (web summary)

Cell shape is determined by a combination of biochemical regulation and mechanical forces. By imaging the dynamic behaviour of growth regulatory proteins in fission yeast and integrating these data within a mechanical model, Abenza *et al*. find that exocytosis plays a dominant role in shaping growth domains.

## Graphical abstract

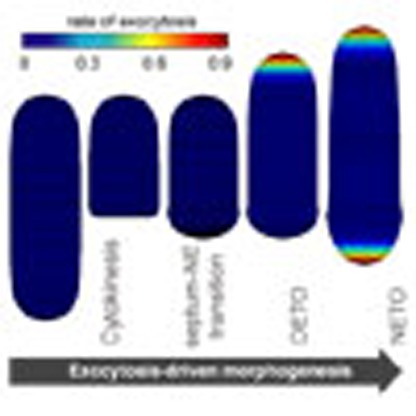

The regular self-assembly of viruses from protein subunits offers an interesting paradigm for how shape can be encoded at the molecular level^1^,^2^,^3^. However, most cells are of a scale that lies above the reach of molecular self-assembly and as a consequence their shape results from a subtle interplay between biochemical regulation and mechanical constraints^2^,^4^,^5^,^6^,^7^.

With their highly regular morphogenesis involving two opposed growth domains, the walled cells of the fission yeast *Schizosaccharomyces pombe* provide a powerful system to address this question^8^,^9^,^10^,^11^,^12^. Following cell division, *S. pombe* cells first grow monopolarly from their ‘old end’ (OE) inherited from their mother but soon thereafter they activate their ‘new end’ (NE) derived from the site of cell septation during an event called New End Take Off (NETO)^8^,^10^,^13^. After NETO, cells grow bipolarly throughout most of the cell cycle until the next cell division, when cells septate giving rise to two similarly sized daughter cells and that re-initiates the morphogenetic growth cycle.

Here we have combined biophysical modelling and quantitative live cell analysis to investigate how the geometry and morphogenetic pattern of fission yeast cells result from the interplay between biochemical and mechanical regulation. We show that neither the global cell shape regulator Cdc42 and its activators Scd1 and Scd2 (refs 14, 15, 16, 17) nor the major cell wall synthesis regulators Bgs1, Bgs4 and Rgf1 (refs 18, 19, 20) are reliable predictors of the geometry of cell growth domains. Surprisingly, we instead demonstrate that their geometry can be defined by cell wall mechanics and the cortical localization pattern of the exocytic factors Sec6, Syb1 and Exo70 (refs 21, 22) across a range of genotypes. By forcefully inducing the re-directioning of exocytic vesicle fusion to broader areas of the cell cortex, we further show that this induces proportional shape changes to growth domains, demonstrating that both features are causally linked. We propose that cell wall mechanics and exocytic pattern suffice to account for growth domain morphogenesis throughout the cell cycle in this species.

## Results

### Growth domains undergo shape changes through the cell cycle

**Figure 1:**
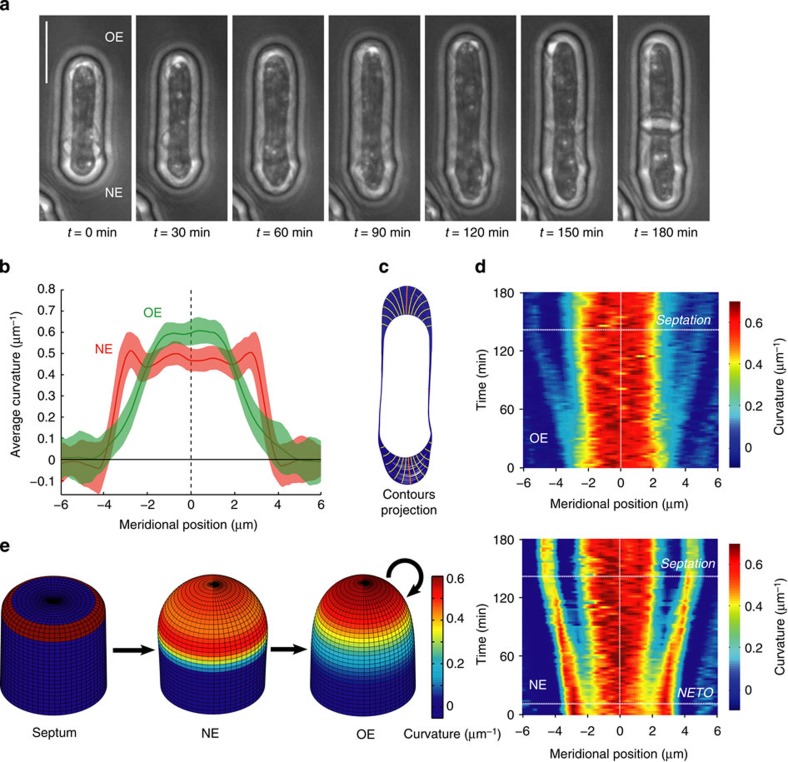
Morphological evolution of cell growth domains in fission yeast. (**a**) Transmitted light images illustrating the changes in OE and NE shape in a fission yeast cell throughout interphase. Left, cell immediately before NETO. Images are maximum intensity projections of z-stacks comprising the most equatorial 2 μm of the cell. Scale bar, 5 μm. (**b**) Average meridional curvature of the NE (red line) and OE (green line). The coloured areas around the averages correspond to the standard deviations. *n*=35 OEs and *n*=35 pre-NETO NEs were averaged. (**c**) Cell contours extracted from the time-lapse sequence in **a**. (**d**) Curvature kymographs showing a pointy OE keeping its curvature (top) and a hemispherical NE evolving into a pointy OE (bottom). The plots, corresponding to the ends of the cell depicted in **a**, display the meridional curvature as a ‘heat map’ during 3 h with a 2-min resolution. One of *n*=25 OEs and one of *n*=18 NEs are shown. (**e**) Schematic illustration of the three morphogenetic transitions observed in *S. pombe*: (*i*) the deformation of the flat post-cytokinesis septum into a hemispherical NE (first arrow), (*ii*) the growth of the hemispherical NE into the pointy OE (second arrow) and (*iii*) the steady growth of the OE (third arrow).

To investigate how fission yeast cells are locally shaped, we quantitated the curvature of their growth domains, which are the areas that undergo geometrical changes through the cell cycle (Fig. 1a and Supplementary Movie 1 and Methods). Although initially flat at septation, we found that the shape of the NE (pre-NETO) becomes roughly hemispherical (Fig. 1b, red). By contrast, we found that the OE displays a much pointier, non-hemispherical shape distinct from that of the NE (Fig. 1b, green). Quantitation of end curvature through time (Fig. 1c) revealed that OE curvature does not change noticeably throughout growth, indicating that OE geometry results from a stable growth domain dynamics (Fig. 1d). On the other hand, NE geometry changes substantially following NETO and continues to change until late G2 phase, when NEs acquire an OE geometry while leaving gradually aside scars—cytokinesis-derived structural deformations of the cell wall^23^ (Fig. 1d). Thus, the morphogenesis of *S. pombe* is characterized by a simple growth domain dynamics according to which NEs transition from flat to hemispherical and then morph gradually into OEs, whereas OEs maintain their geometry that acts as a stable morphogenetic attractor (Fig. 1e).

### A mechanical model of fission yeast cell growth

**Figure 2:**
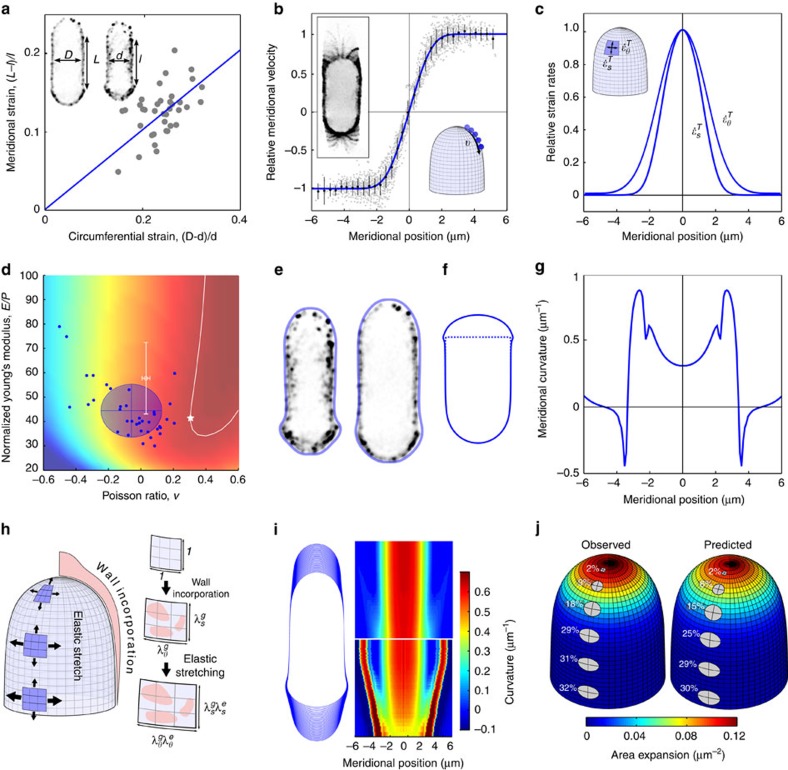
A mechanical model of fission yeast growth. (**a**) Elastic deformation of the cell wall following cell plasmolysis. The diagonal line indicates a ratio of 1:2 between the meridional and circumferential strains. Top: fluorescence of a Qdot-labelled cell before (left) and after (right) plasmolysis (Note: here and in other images contrast has been inverted for clarity). (**b**) Measured wall element displacements at OEs using fluorescent Qdots. Top left: temporal projection of a Qdot-labelled cell during growth. (**c**) Canonical wall expansion profile at the OE. The meridional and circumferential strain rates were inferred from the best fit of the wall displacement field shown in **b**. (**d**) Elastic properties space. Blue dots and ellipse: normalized Young’s modulus (*E/P*) and Poisson’s ratio (*ν*) inferred from the data points in **a**. White circle and error bars: best material properties inferred from the whole-cell simulations of **e** and **f**. Background colour map: fit between the canonical OE expansion profile and the growth simulations (**j**) for each pair [*ν, E/P*] (dark red: best fit). White level curve: sub-region of the space yielding a predicted expansion profile within the 95% confidence of the observed profile (star: point closest to the experimental elastic properties). (**e**) Contour of a plasmolysed cell (left) used as input to the elastic shell model. Numerical inflation of the plasmolysed cell yields a turgid cell geometry (blue) very similar to the observed cell (background: Qdot-labelled cell). (**f**) Simulation of the septum-NE transition with the relaxed septum shown as a dashed line. (**g**) Predicted NE curvature following the septum-NE transition (compare with Fig. 1b). (**h**) Morphogenetic model of cell ends where both wall incorporation and elastic deformation contribute. (**i**) Simulation of cell growth using the experimental wall areal expansion as growth input and mechanical build-up of circumferential anisotropy. Left: schematic of cell end curvature evolution through time at the simulated OE/NE (top/bottom). Right: simulated curvature kymographs for the OE/NE (top/bottom; compare with Fig. 1d). (**j**) Comparison of the canonical expansion profile (left) and the predicted expansion from the simulations (right). The areal expansion profiles (colour map) and expansion anisotropy (ellipses) are predicted precisely by the model.

As a first step to explain the origin and maintenance of the cell ends’ geometry, we developed a technique to label cells with fluorescent quantum dots (Qdots) and use them as fiducial marks to track the cell wall deformation (Supplementary Fig. 1 and Supplementary Movies 2 and 3). We started by measuring the elastic deformation of the cell wall combining our Qdots technique with cell plasmolysis experiments, where we induced cells to lose water and turgor pressure (Supplementary Movie 4). Quantification of Qdot repositioning during plasmolysis revealed large elastic strains in the cell wall reaching as much as 30% (Fig. 2a). Moreover, the elastic stretch in the circumferential direction exceeds the meridional stretch by a factor of two (Fig. 2a). These striking elastic effects confirm that growth domain morphogenesis cannot be fully explained unless wall elasticity and mechanics are taken into consideration. We then used the Qdots technique to characterize the expansion of the cell wall during active growth, by tracking the displacement of wall elements at growing cell ends. Focusing on stably growing OEs, we found that all OEs (*n*=19) share the same characteristic wall displacement field (Fig. 2b and Supplementary Fig. 2). The reproducible morphogenesis of OE allowed us to put forward a canonical wall expansion profile (Fig. 2c). This canonical profile is characterized by a sharp meridional gradient in the meridional 
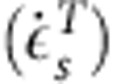
 and circumferential 
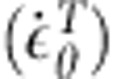
 strain rates, so that more than 90% of wall expansion takes place within 3 μm of the pole. As for the reversible elastic deformation on the cell cylinder (Fig. 2a), wall expansion at the OE favours the circumferential direction (Fig. 2c). To sum up, the glucan wall of *S. pombe* experiences large elastic strains because of the internal turgor pressure, its growth is focused in a narrow area extending ∼3 μm around the OE poles, and its elastic and growth deformations both favour the circumferential direction over the meridional direction (that is, the deformation is circumferentially anisotropic).

We attempted to capture these features using the simplest possible morphogenetic models (Fig. 2d–g). First, we modelled the cell shaft as a cylindrical shell with linearly elastic properties and used the results from the plasmolysis experiments (Fig. 2a) to get an initial assessment of the wall’s elastic properties (Supplementary Note). This analysis yielded a rather broad distribution of elastic parameters with a mean Young’s modulus to turgor pressure ratio (*E/P*) of 44 and a mean Poisson’s ratio (*ν*) of −0.06 (Fig. 2d). Given a turgor pressure of *P*=1.5 MPa (note added in proof of ref. 24), our best estimate of the Young’s modulus of the cell wall is 66 MPa. To test more precisely the validity of these material properties, we developed a model taking into account the precise cellular geometry. Based on recent models^25^,^26^,^27^ and the uniform composition of the cell wall^9^,^28^,^29^, we simulated the cell as a thin elastic shell with homogeneous and isotropic elastic properties (Supplementary Note). Using the plasmolysed cell geometry as initial conditions, we inflated the cell and assessed which set of material properties allowed the simulated deformed shape to best fit the observed turgid cell geometry. Despite the simplicity of the model, it reproduced the deformation of plasmolysed cells accurately (Fig. 2e) while also giving robust estimates of the elastic material properties (Fig. 2d), specifically 58 for the Young’s modulus to turgor pressure ratio (*E/P*) and 0.03 for the Poisson’s ratio (*ν*). As a final test of this model, we also attempted to predict the abrupt morphogenetic transition associated with the deformation of the flat septum into the near hemispherical geometry of the NE (Figs 1e and 2f). This phase of morphogenesis presents a new challenge because the resting length of the septum is unknown, as it is first formed within the confine of the load-bearing wall of the mother cell and therefore does not experience any deformation until the daughter cells have separated. However, we have found realistic NE morphologies for a broad range of resting lengths as long as these exceeded the observed septum length by ∼20% (Supplementary Note). Using the same elastic model as before and a resting length of 1.3 for the septum, we were able to reproduce the morphogenetic transition precisely, including the appearance of the division scar (Fig. 2f,g) and the characteristic meridional curvature of the NE (compare Figs 1b and 2g).

The ability of the elastic shell model to reproduce the morphological changes associated with plasmolysis and septation leaves little doubt about the importance of wall mechanics in shaping these processes. However, it could be argued that the time scale of these morphological changes is so short as to leave no room for any cellular response other than a mechanical response. In contrast, growth domain morphogenesis takes place over a few hours leaving plenty of time for active biological control over this process. We therefore asked whether mechanics remains relevant for those processes and, if so, how biochemical regulation and mechanics are integrated. We propose a model whereby wall elasticity and wall incorporation contribute in parallel to growth domain morphogenesis (Fig. 2h and Supplementary Note). Our model posits the existence of a relaxed, stress-free cell whose geometry may depart significantly from the turgid cell geometry (as seen in the plasmolysis experiments of Fig. 2e). This relaxed geometry, however, is also subjected to growth by incorporation of new wall material. Thus, the characteristic wall expansion profile made visible by the Qdots (Fig. 2b) is the by-product of a biochemical process controlling the incorporation of new wall material and a mechanical process whereby the wall is stretched elastically as it experiences the internal turgor pressure of the cell. To test how such a model can account for the measured canonical wall expansion profile, we modelled cell growth using the observed areal expansion as growth input (the areal expansion is the sum of the meridional and circumferential strain rates of Fig. 2c). Both the geometry of the OE and the anisotropy of its wall expansion were reproduced accurately with this model (Fig. 2i,j). Furthermore, using the same wall incorporation profile as before, the growth model also successfully predicted the evolution of the NE geometry observed after NETO (Fig. 2i). Thus, our morphogenetic model offers a simple, self-consistent mechanism for how wall assembly and elasticity combine to create cell end shape throughout the cell cycle in fission yeast. Finally, the growth model provides us with a third method to get at the elastic properties of the cell wall, albeit now looking at the long-term elasticity of a growing cell wall. We therefore explored what values of the Young’s modulus to turgor pressure ratio and Poisson’s ratio permit the most accurate prediction of the expansion profile. We found a broad domain of material property values compatible with the 95% confidence intervals observed for the wall expansion profile (Fig. 2d). Within this domain, we selected the material properties falling closest those obtained in the plasmolysis experiment (*E/P*=40 and *ν*=0.3).

### Morphogenetic potential of cell end-distributed machineries

**Figure 3:**
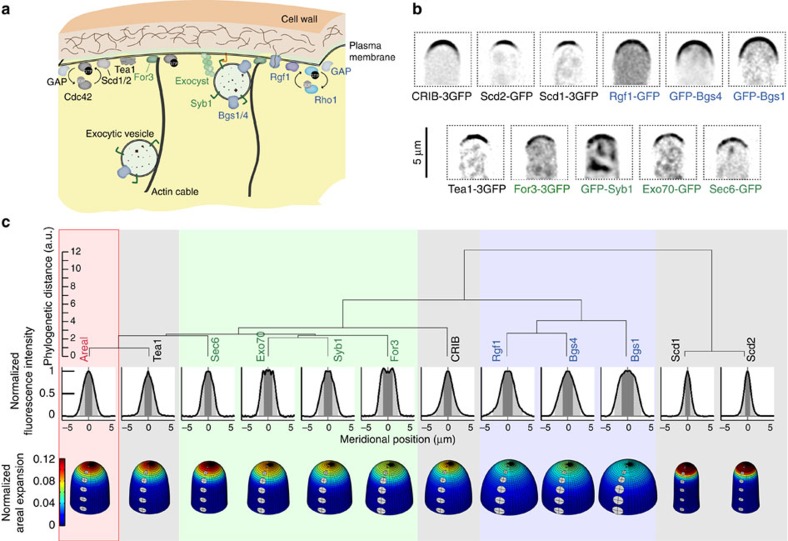
Morphogenetic potential of cell end-distributed factors. (**a**) Diagrammatic representation of the function of 11 key factors involved at different levels of the polarized growth cascade (black, polarity factors; green, exocytosis; blue, glucan synthesis). (**b**) Cortical OE distribution *in vivo* of the factors fluorescently labelled. The images are OAI sum projections (12 frames every 10 s). (**c**) Top panels: The symmetrized average OE distribution of each GFP-labelled marker (*n*=29). The darker grey area corresponds to the full-width at half-area (FWHA), a parameter quantifying the spread of a given marker in the apical membrane; the lighter grey area corresponds to the full-width at 95% area (FW95A), which defines the shape of the distribution along the membrane (see Supplementary Fig. 3 for the distributions’ standard deviations). Bottom panels: Modelled OE morphologies obtained when using the average factor distribution as a proxy for new wall incorporation. Note how different factors belonging to similar machineries have similar distributions and lead to similar geometries, as quantitatively illustrated by their respective branch length in the dendrogram. The Areal distribution is inferred from the strain rate profiles of Fig. 2.

Because of its ability to predict OE and NE morphogenesis, we surmized that the measured areal wall expansion profile (referred to as Areal thereafter) could be a good candidate function for the distribution of a growth-controlling molecule. Any molecule presenting the same meridional distribution could be integrated in our model and predict growth domain morphogenesis to the same degree of accuracy as what was achieved in Fig. 2i,j. We therefore looked for such a molecule(s) by quantitating the cortical OE distribution of three different types of green fluorescent protein (GFP)-labelled machineries known to be involved in the polarized growth cascade (Fig. 3a,b and Supplementary Fig. 3a,b)^12^,^13^,^22^,^30^: (i) polarity factors, specifically the upstream polarity landmark Tea1, the global shape regulator Rho-like GTPase Cdc42 (visualized indirectly in its GTP-bound state by the localization of a fluorescently labelled CRIB domain, whose distribution has been shown to mirror that of Cdc42 (ref. 31), its guanosine exchange factor (GEF) Scd1 (ref. 16) and the Scd1 co-factor Scd2 (ref. 17); (ii) the exocytic machinery, specifically the actin cable nucleating-formin For3 (ref. 32), the synaptobrevin homologue, the v-SNARE Syb1, a general exocytic vesicle marker^22^, and Sec6 and Exo70, two subunits of the exocyst, a tethering complex essential for the exocytic vesicles docking at the plasma membrane^21^, and (iii) the cell wall synthesis machinery, specifically the GEF Rgf1, a co-factor of the β-glucan synthase regulator Rho1 (ref. 19), and the β-glucan synthases Bgs1 (refs 2, 4, 5, 6, 7, 18) and Bgs4 (refs 8, 9, 10, 11, 12, 20).

Averaging of the cortical distribution of the factors across many cells allowed us to establish a ‘canonical’ distribution for each factor, which could then be compared among themselves (Fig. 3c middle row, and Supplementary Figs 3b and 4). A first important conclusion of this comparative analysis of marker distribution is the ‘phylogenetic’ clustering of factors belonging to the same machineries (Fig. 3c top row dendrogram, and Supplementary Fig. 3c–e). In particular, exocytosis markers and glucan synthesis markers form two non-overlapping groups in terms of apical distribution and therefore validate our hypothesis that the relative importance of competing morphogenetic pathways can be ascertained by these methods. Interestingly, comparison between the canonical distributions and the Areal profile revealed that CRIB, Tea1 and exocytosis-associated factors are closest among themselves and to the Areal profile, whereas cell wall synthesis-related factors and the Cdc42 activators are most distant (Fig. 3c top row, and Supplementary Fig. 3b–e; note: we did not pursue further analyses of Tea1 because of the extremely high standard deviation of its distribution among the population of OEs, see Methods for details). This conclusion was also supported by simulations where each canonical distribution was used as a proxy for wall incorporation (Fig. 3c bottom row): in general, exocytosis-related factors led to cell ends of the right width and curvatures, whereas Cdc42 activators led to much narrower cell ends, and glucan synthesis-related proteins led to much wider cell end geometries. Thus, our analysis of the factors’ canonical distributions generally suggested that the exocytic machinery could be the direct determinant of growth domain establishment and morphology, while instead the Cdc42-activating machinery and glucan synthesis factors might not. It also established that two numbers inferred for the fluorescence distribution—the full-width at half-area, FWHA, and the full width at 95% of the area, FW95A—are valid predictors of the morphogenetic potential of the distribution.

A potential caveat of this analysis is the assumption that the recorded fluorescence profiles are accurate representations of the activity of the factors and of their morphogenetic functions. Although this assumption is likely to be valid for factors acting directly on wall deposition such as the glucan synthases Bgs1 and Bgs4, its validity is less clear for factors far upstream of wall assembly such as Cdc42. Indeed, we were able to make many markers better predictors of morphogenesis by simply postulating a nonlinear readout of the fluorescence profile and some degree of meridional advection of the profile (Supplementary Fig. 5). We reasoned that a more robust assessment of the importance of different classes of factors in shaping the growth domains could be obtained by exploiting the powerful genetic toolbox of *S. pombe*, looking for genotypic conditions that induce changes in the distribution of markers and/or in the morphogenesis of the growth domains. A factor both necessary and sufficient for growth domain morphogenesis ought to correlate systematically with the observed cell end geometry across different genotypic conditions.

### Neither Cdc42 nor cell wall synthesis reliably predict growth pattern

**Figure 4:**
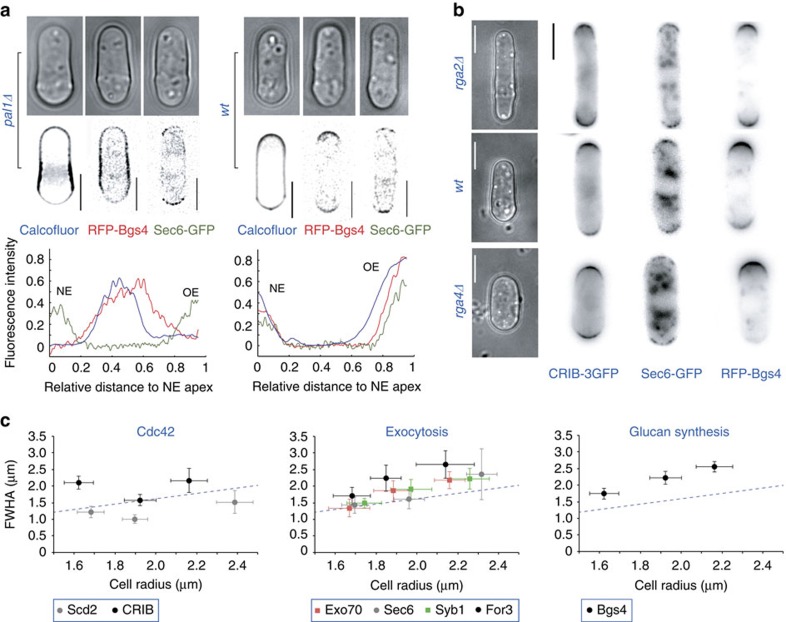
Wall mechanics and exocytosis suffice to drive growth domain morphology. (**a**) Top: Sum intensity projection images of *pal1**Δ*** and wild-type cells stained with calcofluor white, which binds to the linear chains of β-1,3 glucan, or expressing RFP-Bgs4 and Sec6-GFP. Scale bars, 5 μm. Bottom: Quantitation of the fluorescence intensity of each of the three reporters in *pal1**Δ*** and wild-type. (**b**) Sum intensity projection images showing *rga2*Δ (top), wild-type (middle) and *rga4*Δ (bottom) cells expressing CRIB-3GFP, Sec6-GFP or RFP-Bgs4. Scale bars, 5 μm. Transmitted light images of the mutants and wild-type are depicted on the left. (**c**) Plots illustrating the average full-width half-area (FWHA) of the distribution of Cdc42 markers (CRIB-3GFP and Scd2-GFP; left), exocytosis markers (Sec6-GFP, Exo70-GFP, For3-3GFP and GFP-Syb1) and the glucan synthesis marker RFP-Bgs4 against the cell width in the mutants *rga2*Δ (left within each plot) and *rga4*Δ (right within each plot) and the wild-type (middle within each plot). *n*=26 cells per condition. The standard deviations are represented as crosses emerging from each average value (coloured shapes). The dashed line corresponds to the observed relationship between the FWHA of the wild-type wall expansion strains and the cell radius.

To ask whether the glucan synthesis machinery could underlie growth domain morphogenesis, we used *pal1***Δ** cells. Pal1 (refs 8, 10, 13, 33) is a membrane-associated protein that interacts and co-localizes with the endocytic adaptor Sla2p/End4p^14^,^15^,^16^,^17^,^34^ and is involved in cell morphogenesis and cell wall integrity. We found that in *pal1***Δ** cells glucan synthesis factors become delocalized around the cell periphery, yet cells maintain their ability to direct exocytosis to cell ends and to grow cylindrically (Fig. 4a, Bgs4 delocalization shown; Dodgson, Chessel *et al*., manuscript in preparation). Therefore, a properly localized glucan synthesis machinery is not a necessary condition for growth domain morphogenesis, consistent with previous evidence^18^,^19^,^20^,^35^.

To ask whether the Cdc42 machinery could directly underpin cell end growth pattern, we quantitatively compared the average extent of its localization at cell ends with the average measured width of cell growth domains. In previous studies, this type of comparison has led to the proposal that the extent of Cdc42-GTP localization at cell ends controls global cell geometry by modulating cell end width^14^,^15^,^21^,^22^,^36^. To test this idea further, we did this analysis not only in wild-type cells but also in narrower *rga2***Δ** cells (Rga2 is the GTPase-activating protein (GAP) for Rho2, another GTPase is involved in polarized growth^23^,^37^,^38^,^39^,^40^) and in wider *rga4***Δ** cells (Rga4 is the major Cdc42 GAP^14^,^15^,^36^,^37^,^39^), to explore a wider range of phenotypic variety. To our surprise, we found that the cortical extent of the Cdc42 machinery and cell width do not correlate across those morphologically diverse genotypes (Fig. 4b,c, CRIB and Scd2 FWHAs shown, and Supplementary Fig. 6), although there was a good correlation for the exocytic and cell wall synthesis machineries (Fig. 4b,c and Supplementary Fig. 6). Thus, despite the fact that it is well known to be a key determinant of global cell shape^14^,^15^,^25^,^26^,^27^,^35^,^36^, GTP-Cdc42 cortical pattern likely does not directly underpin the specific geometry and growth pattern of OEs.

### Growth domain geometry can be defined by the pattern of exocytosis

Taken together, these observations suggested that it is the pattern of exocytosis that defines the morphogenesis of growth domains. Given that exocytosis has been extensively shown to be a complex process composed of separable events that ultimately lead to the fusion of vesicles with the plasma membrane^9^,^28^,^29^,^41^,^42^, we investigated the possible contribution to growth domain morphogenesis of the sub-pathways controlling where vesicle fusion takes place: actin cables and the exocyst^12^,^13^,^22^,^30^,^43^,^44^. We did this by using the deletion mutant *for3*Δ, which lacks actin cables^31^,^32^. Although slightly misshapen, *for3*Δ cells localized the exocyst subunits Exo70-GFP and Sec6-GFP and the v-SNARE Syb1 at the cell ends (Supplementary Fig. 7). Importantly, the distribution of those proteins over the OE contour was very similar for all three proteins (note that GFP-Syb1, unlike in wild-type cells, localized to the plasma membrane in exocyst-like clusters in *for3*Δ cells; Supplementary Fig. 7). Crucially, like in wild-type cells, there was a strong correlation between the proteins’ localization profiles and the shape acquired by the OEs in *for3*Δ (Supplementary Fig. 7). Thus, we conclude that it is the pattern of exocytosis in general, which is ultimately formed where the exocyst and the membrane fusion machineries are present and active, that might dictate growth domain shape.

### Exocytic pattern and growth domain morphogenesis are causally linked

**Figure 5:**
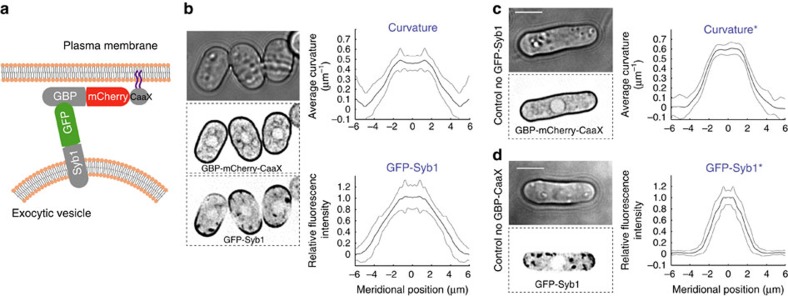
Exocytosis pattern and growth domain morphogenesis are causally linked. (**a**) Cartoon describing the strategy chosen to re-direct GFP-Syb1-containing vesicles to the whole extension of the GBP-mCherry-CaaX-containing plasma membrane. (**b**) Forced re-directioning of exocytic vesicle fusion to broader areas of the plasma membrane causes proportional morphological changes to growth domains. Left: Images showing misshapen cells because of the ectopic distribution of GFP-Syb1 in a GBP-mCherry-CaaX background. Right: Plots depicting the average (thick line) curvature and GFP-Syb1 apical distribution of *n*=30 OEs (the thin lines represent the standard deviation). (**c**) Images showing the distribution of GBP-mCherry-CaaX in the absence of GFP-Syb1 (left) and the canonical curvature of wild-type OEs (right; this corresponds to the data displayed in Fig. 1b; *n*=29 OEs). (**d**) Images showing the distribution of GFP-Syb1 in the absence of GBP-mCherry-CaaX (left) and the canonical OE distribution of GFP-Syb1 in wild-type cells (right; this corresponds to the data displayed in Supplementary Fig. 3; *n*=29 OEs). Scale bars, 5 μm.

To test whether exocytosis is sufficient to specify cell end geometry and growth pattern, we forced the displacement of exocytosis to other areas of the cell cortex using the GFP-GBP (GFP-Binding Protein) system^16^,^45^,^46^ (Fig. 5a). In cells co-expressing GBP-CaaX-mCherry—a GBP-tagged membrane targeting CaaX domain that localizes everywhere on the cell cortex—and the v-SNARE GFP-Syb1, we found that Syb1 was redistributed along broader areas of the cell cortex, sometimes spanning the entire perimeter of the cell (Fig. 5b–d, images), indicating that Golgi-derived exocytic vesicles were fused to the plasma membrane in ectopic places. Strikingly, we found that this led to a proportionate and dramatic change both in the extent and in the shape of the growth domains (Fig. 5b–d, quantitations). This demonstrates that the pattern of exocytosis causatively determines the local geometry of growth domains.

A consequence of this conclusion is that, if the transition from NE to OE geometry following NETO is driven by exocytosis, the pattern of exocytosis should be similar in NEs and OEs. As predicted, when we measured experimentally the cortical distribution of the exocytic proteins Sec6 and Syb1 in NEs undergoing NETO, we found that they are indistinguishable from those in OEs (Supplementary Fig. 8), implying that there is no obvious geometrical feedback into the localization of exocyst and that it is the exocytic pattern that drives both the morphological transition from NE to OE and the stable maintenance of OE geometry throughout cell growth.

### A biomechanical model of fission yeast morphogenesis

**Figure 6:**
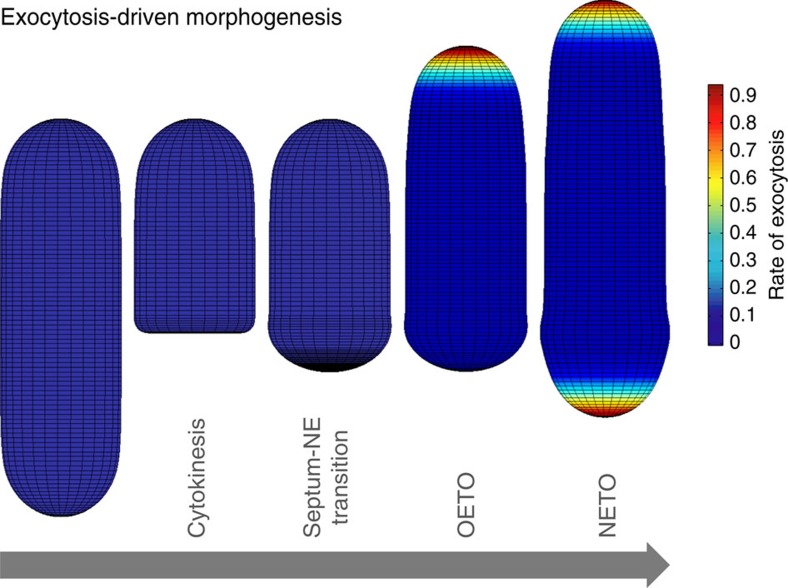
A biomechanical model explaining the morphological evolution of fission yeast cell growth domains through the cell cycle. Cartoon summarizing the dual control of cell growth domain morphology by cell wall elasticity and Sec6-driven exocytosis through the cell cycle.

Altogether, we conclude that cell wall mechanics and the pattern of exocytosis drive growth domain morphogenesis through the entire cell cycle in this species. A schematized model summarizing our findings is shown in Fig. 6 (see also Supplementary Movie 5 for details). During division, when a fission yeast cell undergoes septation at the cell middle it generates two daughter cells, each of which initially possesses one OE-shaped cell end and one flat NE that becomes hemispherical as a result of intracellular pressure and the elastic properties of the cell wall (one daughter cell shown in Fig. 6, left). As each daughter cell starts growth, its exocytic pattern is re-established at the OE triggering monopolar cell growth (Fig. 6, middle). Subsequently, at NETO a stable exocytosis pattern is established also at the NE (Fig. 6 right), which—constrained by the mechanics of the cell wall—gradually changes shape throughout interphase until it takes the characteristic pointy shape of a growing OE, re-initiating the morphogenetic cycle.

## Discussion

Although with our current approach we cannot definitively assert that Cdc42 activation and glucan synthesis are not directly required for growth pattern, our data suggest that neither the Cdc42-activating machinery nor the cell wall synthesis machinery are good causative predictors of that pattern. In the case of glucan synthases, our results with *pal1***Δ** cells demonstrate that they are not causative for growth domain morphogenesis. This could indicate that, although the synthesis of glucans is essential (as they are the main component of the fission yeast cell wall), the changes that the cell wall undergoes during cell growth do not only rely on that process, but also on other molecular activities that define the extent of glucan synthesis—or its remodelling—in different locations of the cell cortex and that do this in an exocytosis-dependent manner. Some candidate machineries are glucanases, glucanosyl transferases and other enzymes that modify the glucans and the rest of components of the cell wall, like mannans^17^,^28^,^47^,^48^, which could be studied in the future if the challenge of tagging and studying the fine localization of proteins acting between the plasma membrane and the cell wall is overcome.

Surprisingly, our results also imply that, although Cdc42-GTP is known to recruit exocytic factors and to help drive exocytosis^30^,^31^,^32^,^49^,^50^, the precise pattern of growth is defined by the subsequently established pattern of exocytosis, and not by the original pattern of Cdc42 activity. Thus, although Cdc42 is clearly implicated in global cellular morphogenesis^14^,^22^,^50^,^51^,^52^,^53^, its role might be primarily at the signalling level upstream of morphogenesis, whereas exocytosis might act as the downstream effector that modulates locally cell geometry.

In fission yeast, it is broadly agreed that exocytosis is determined mostly by the polarized transport of vesicles from the Golgi to cell ends via actin cables and their reception by the exocyst (a multiprotein tethering factor) with both processes considered independent and simultaneous^21^,^30^,^44^,^50^. However, the contribution of each of those sub-processes in determining the overall pattern of vesicle fusion with the plasma membrane and, consequently, in the establishment and maintenance of cell geometry, is not clear. On the one hand, the exocyst is able to reach cell ends in the absence of actin cables and it is essential for fission yeast viability^19^,^21^,^43^,^44^. This could be taken to suggest that the exocyst is the major determinant of cell growth and, hence, of cell geometry. However, with the data available it cannot be concluded that the essential role of the exocyst is exclusively derived from its direct role in exocytic events. For example, recent data support a role for Sec3 as coordinator of actin cable assembly and actin patch internalization^44^, which would imply that the role of the exocyst as a morphogenetic factor could go beyond its tethering function. On the other hand, although the exocyst can reach the cell ends without actin cables, in wild-type cells it appears to be assembled and directed to the cell membrane through them^43^,^44^. This makes dissecting the network that leads to exocytosis very complicated. In this regard, we believe our finding that the pattern of growth is modulated by exocytosis will help shift the focus of future morphogenesis studies from the role of Cdc42 activity and the processes it directly controls, to the role of the specific processes controlling tethering and fusion of vesicles with the plasma membrane.

In our biophysical model, for simplicity and because of insufficient information in the literature about the different factors examined, we did not incorporate any information about the factors’ recycling dynamics (binding/unbinding at the plasma membrane, diffusion at the membrane and the cytoplasm, etc) or other potentially nonlinear interactions at the cell cortex, as has been done, for example, in previous modelling work focusing on the role of Cdc42 in cell polarization^26^,^54^,^55^. Here, we assumed that the spatial distributions observed for the different factors from populations of cells were reflective of a steady-state implicitly containing all relevant nonlinear interactions, and instead we focused on investigating whether those distributions were good predictors of growth pattern. The finding that the localization pattern of exocytic factors, but not of other factors, correlates with growth pattern across a range of genotypes and modelling parameters, and the fact that relocalization of the exocytic machinery in cells causatively relocalizes and alters growth, indicates that the simpler model recapitulates the relevant biology at play.

Future biophysical and modelling efforts will be needed to address in detail the relevance of those recycling dynamics to the fine spatial and temporal modulation of cell growth, in this and other cellular systems.

## Methods

### Strains, media and image acquisition

The double fluorescently marked strains and the strains containing the deletions *pal1*Δ, *rga2*Δ and *rga4*Δ (which derived from the commercially available ‘*S. pombe* Haploid Deletion Mutant Set version 2.0’ strains collection (Bioneer Corporation; http://pombe.bioneer.com)^30^,^56^) as well as the GBP-mCherry-CaaX GFP-Syb1 strain were generated following standard crossing methods^35^,^57^. Supplementary Table 1 contains a list of all the strains used in this study.

Cells were grown exponentially at 32 °C during 48 h in rich YES (yeast extract with supplements) medium before specific treatments or imaging. For this purpose, we used 35 mm glass bottom plates (MatTek Corporation) or eight-chambered coverglass Lab-Tek II plates (Nunc, Thermo Fisher Scientific Inc.) pre-coated with lectin (1 mg ml^−1^; Sigma; L1395 and Patricell Ltd; L-1301-25). A DeltaVision system (Applied Precision/GE Healthcare) comprised of an Olympus IX81 epi-fluorescence inverted microscope, Olympus UPlanSapo × 100 and × 60 oil immersion lenses (numerical aperture 1.4 and 1.42, respectively, Olympus) and 1.512 refractive index immersion oil (Applied Precision) was used for imaging through the proprietary software SoftWoRx.

Live calcofluor white (Fluka, Sigma-Aldrich Co. LLC) staining was performed by incubating cells in 1/100 (v/v) calcofluor white in YES. Cells were then imaged using a DAPI filter set. Treatment with hydroxyurea (HU) was carried out culturing the cells during 3 h in 0.025 M of this drug in YES medium before imaging.

Qdots (Qdot 605 Streptavidin Conjugate; Life Technologies) were imaged with a standard TRITC (Tetramethylrhodamine; 555/28 617/73) filter set, whereas cells expressing GFP–, red fluorescent protein (RFP)– or mCherry–tagged proteins were imaged with fluorescein isothiocyanate and TRITC filter sets. All fluorescence imaging was performed with the DeltaVision tool 'Optical Axis Integration' (OAI). The bright-field images were acquired as z-stacks comprising 5.4 μm (separation between z-planes: 0.2 μm). Image processing was done using SoftWoRx, the open-source software Fiji (http://fiji.sc/wiki/index.php/Fiji) and Matlab (MathWorks). Two-dimensional (2D) or three-dimensional deconvolution was used only for illustration purpose. Apart from very few exceptions, Qdots were not observed to detach from the cell wall throughout the times of observation.

### Kinematic analysis

Our empirical analysis of cell morphogenesis is articulated around three axes of quantification: (i) the change in cell geometry through the cell cycle, (ii) the deformation of wall elements during growth and (iii) the distribution of cortical markers putatively involved in controlling growth. The result of these quantification steps is three functions, which are the basis of all further analyses. These functions are: the meridional curvature (*κ*_*s*_), the meridional velocity (relative displacement) of wall elements (*v*) and the fluorescence intensity profile of cortical markers (*γ*), which are all functions of the meridional distance from the pole of the cell (*s*). All quantification tasks were performed with newly developed computer tools written with Matlab (MathWorks). All programmes are freely available on request.

(i) Quantification of cell geometry: To study the evolution of the curvature of wild-type cells during the cell cycle, bright-field z-stacks encompassing a thickness of 5.4 μm (separation between z-planes: 0.2 μm) were acquired each 2 min during 4 h (25 cells; Supplementary Movie 1). Subsequently, maximum intensity projections of the z-stacks were used to define one bright-field image for the cells at each time point. The outline of growing cells was then determined from these bright-field images using computer-assisted tools. The initial step requires the user to select points around the contour of the cell on the first image of a time-lapse sequence, using the zone of high contrast associated with the cell wall (Supplementary Fig. 1a). The programme then uses a spline interpolation of the initial guess to position *n* uniformly spaced points (typically *n*=60) delineating the cell contour. To maximize the fit between the contour and the bright-field image, the *n* anchor points of the contour were moved by small steps orthogonal to the curve while computing the integral of the pixel intensity around the entire cell contour. Steps that increased the integral were kept while others were rejected. The contour of the cell at a given time point was then used as the starting contour for the next time point. This algorithm led to a robust tracking of the cell geometry (Supplementary Fig. 1a).

The kinematic analysis was based on a so-called Eulerian specification of cell morphogenesis, according to which all variables are evaluated at fixed positions on the cell surface. As morphogenesis in *S. pombe* is confined to the NE and OE of the cell, a natural Eulerian point of reference is the pole of the NE and OE. In that context, the pole was defined as the longest orthogonal path connecting the first and last cell outlines in the time-lapse sequence (Supplementary Fig. 1b). All variables such as curvature and the fluorescence intensity of cortical markers were measured and, ultimately, averaged at fixed meridional distances (*s*) from the pole of the cell. On occasion, asymmetry in the cell contour led to spurious pole positioning. The misplaced pole was then shifted ‘manually’ so as to maximize the symmetry of the curvature and fluorescence profiles. The measurement of kinematic variables such as curvature was performed at a fine spatial resolution by resampling the cell contour on either side of the pole with 900 points spaced by 0.1 pixels (6.7 nm). This fine subdivision of the cell contours was used for computational purposes (for example, to improve the accuracy of integration schemes) and is not meant to represent the resolution of the microscope setup.

The meridional curvature was calculated as *κ*_*s*_(*s*)=*α*(*s*)/*l*, where *α* is the angle measuring the amount of rotation between two successive contour segments and *l*=6.7 nm is the length of those segments. Curvature kymographs were prepared by treating the curvature matrix for a time-lapse sequence as an image where pixel intensities are set by the magnitude of the curvature for each meridional and temporal positions (Supplementary Fig. 1c). The measured curvature vary temporally and is not perfectly symmetrical with respect to the pole (that is, *κ*_*s*_(*s*)≠*κ*_*s*_(−*s*)). The growth domain geometry was determined by averaging the meridional curvature at fixed meridional distances from the pole of the cell. The curvature was also made symmetrical by averaging the ‘left’ and ‘right’ side of the cell as defined by the position of the pole. For OE growth domains, the averaged symmetrical curvature fits closely the observed curvature (Supplementary Fig. 1d). The geometry of the NE changes abruptly after NETO and therefore could not be computed by averaging entire time-lapse sequences. Instead, a total of 35 recently divided cells were used to define canonical NE geometry.

(ii) Quantification of wall deformation using Qdots: To track the cell wall deformation during apical expansion, we tagged exponentially growing cells with streptavidin-conjugated Qdots via biotinylated lectins (isolectin GS-IB4 from *Griffonia simplicifolia*, biotin-XX conjugate; Invitrogen), which act as molecular bridges between nanoparticles and the cell wall, as described in Abenza *et al*.^5^,^45^ (Supplementary Fig. 1f). Tagged cells were imaged every 10 min for 3 h at room temperature in both transmitted light and the TRITC fluorescence channel using the DeltaVision specific tools OAI and 2D-deconvolution (Supplementary Movies 2 and 3).

The faint and variable fluorescence of Qdots prevented us from implementing an automatic tracking protocol. Instead, their trajectories were identified manually from maximum intensity projection images of the fluorescence channel (Supplementary Fig. 1e–h). These images were imported in Inkscape (GNU general public license) and each Qdot path overlaid with a Bézier curve. The parameters for the Bézier curves were then extracted from the scalable vector graphics (.svg) files and imported directly in Matlab. The intersections between a Qdot path and the cell contours were taken as the position of the Qdot and the wall element to which it is bound.

One challenge for the quantification of wall growth in *S. pombe* is the variable nature of the growth increments at the cell ends. To circumvent this problem, we focused our analysis on the relative meridional displacement of wall elements with respect to a given growth increment of the cell end (Δ*h*). If the growth domain geometry remains constant during morphogenesis, then any displacement observed at the pole of the cell must correspond to the total growth incurred along the cell’s meridian, that is, the pole is simply pushed forward by the growth occurring below it. For a Qdot moving from position *s* to position *s*+Δ*s* during the growth interval, the relative displacement is v=Δ*s*/Δ*h*. The relative displacement of Qdots was fitted with the function *v*(*φ*)=*sinφ* (*a*+*bφ*^2^+*cφ*^4^), where *φ* is the angle of the normal to the cell contour. In fitting, the relative displacement, the constraint: *v*(π/2)=*a*+*b*(π/2)^2^+*c*(π/2)^4^=1 was imposed; that is, the relative displacement was normalized to 1 at the equator (*φ*=π/2).

(iii) Quantification of fluorescently tagged proteins: We studied the OE localization of different proteins—constituents of distinct intracellular machineries—that have been reported to occupy the growing cell domes: Tea1, For3, CRIB, Scd1, Scd2, Syb1, Sec6, Exo70, Rgf1, Bgs1 and Bgs4 (refs 9, 15, 17, 18, 19, 20, 22, 30, 32, 37, 43). To correlate the distribution profiles of these proteins at the cell ends with the canonical pattern of cell wall strains observed during growth, we imaged cells expressing GFP–Bgs4, GFP–X or both RFP–Bgs4 and X–GFP (or GFP–X, depending on the protein; 'X' being one of the proteins mentioned above) with the ‘OAI’ mode in the fluorescein isothiocyanate and/or TRITC fluorescence channels. Considering the facts that: (i) potentially, each protein would behave differently in terms of aggregation and dynamics (for three examples, see refs 15, 30, 45) and (ii) the OE curvature remains quite constant in 2 min intervals (Fig. 1d), all time-lapse imaging of fluorescently labelled proteins was performed every 10 s for a total of 2 min for each cell analysed (*n*=29–35 cells, depending on the strain).

The fluorescence profile of the proteins was obtained by integrating the pixel intensity within a narrow window centred on the cell online (Supplementary Fig. 1i). The intensity of all the frames was averaged and used for subsequent analysis (see Supplementary Fig. 3b for details). The averaged fluorescence profile was fitted with the smoothing spline function *csaps* of Matlab using a parameter value of 0.8, which corresponds to a weak smoothing. Background subtraction and normalization were performed by subtracting the minimal intensity value of the spline and then scaling the profile so that the maximal fluorescence intensity is 1.

As an example, at the resolution at which we performed our experiments, in the 10 min timeframe of filming 3–4 main exocytic patches were detected on average, as can be observed in the Sec6 panel of Supplementary Fig. 4. As shown in that figure, and described in the text, this was deemed sufficient to assume ergodicity, that is it was sufficient to claim that the distribution of the exocytic reporter at the single-cell level was reflective of the average distribution of that reporter at the population level.

The calculation of the FWHA (Supplementary Fig. 4a) and FW95A of the fluorescence profiles was performed using a customized Matlab routine. For the FWHA, the routine divides vertically the area under the curve of each distribution profile in three parts: a central and two lateral parts, in a way that the sum of the two lateral parts is equal in area to the central one. Then, the width of that central part is computed as the FWHA.

For the *pal1*Δ experiments, we computed the fluorescence intensity of the markers around the entire cell contour. We used only wild-type-like cells for our analysis (as reported, this mutant displays a variety of shapes: stubby, pear-like, lemon-like and wild-type-like^33^).

The kinematic analysis described above yields three variables: the meridional curvature (*κ*_*s*_(*s*)), the meridional velocity of Qdots (*v*(*s*)) and the fluorescence profile of cortical markers (*γ*(*s*)). From these basic variables, it is possible to compute many additional descriptors of morphogenesis.

The *cell geometry* was computed from the average meridional curvature (*κ*_*s*_) using the equations:




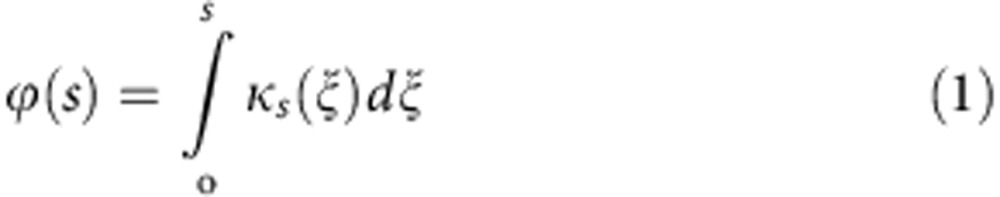







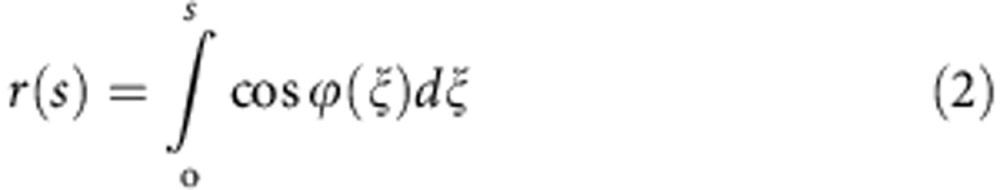







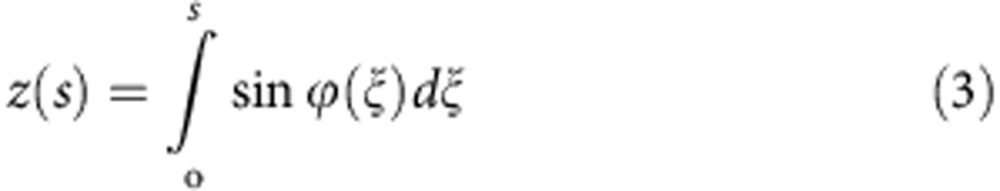







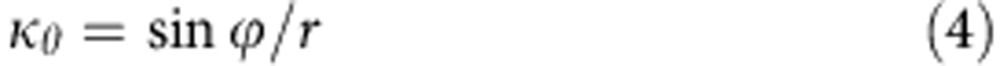




where *κ*_*θ*_ is the circumferential curvature and *ξ* is a dummy variable of integration.

The local rates of wall expansion or *strain rates* are given by the relations:




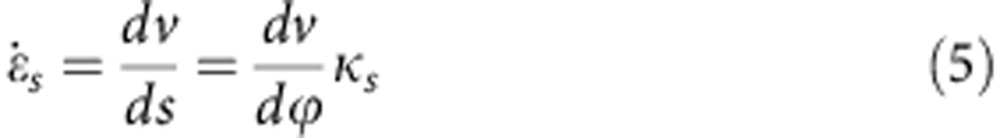







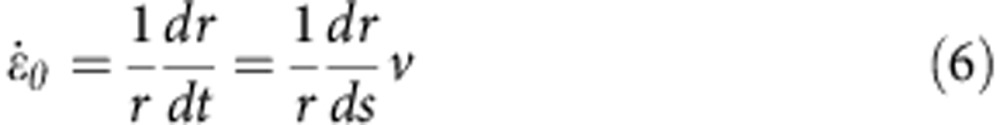




The two strain rates are more easily interpreted in terms of the areal strain rate 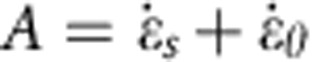 and the deformation anisotropy α=(*w*-*l*)/*w*. Here *w* and *l* represent the evolving width and meridional length of a small wall element as it is displaced away from the pole. If the wall element is found initially near the pole at position *s*_*0*_ and has circular geometry *w*_*0*_=*l*_*0*_, then its dimensions as it migrates along the meridian will be: *w*(*s*)=*w*_*0*_*r*(*s*)/*r*(*s*_*0*_) and *l*(*s*) such that *t*(*s*)-*t*(*s+l*(*s*))=*t*(*s*_*0*_)-*t*(*s*_*0*_+*l*_*0*_), where *t*(*s*) is




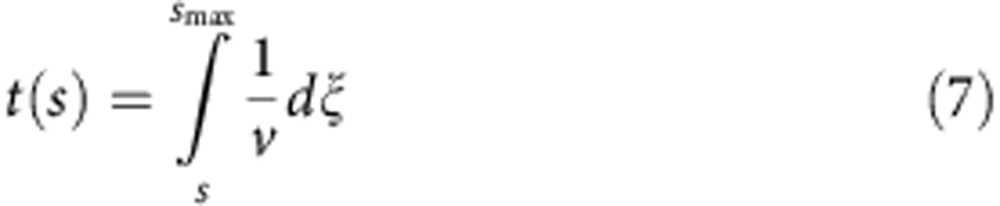




According to our model (see Supplementary Note for details), the areal strain rate (*A*) reflects closely the rate of wall incorporation and thus it is a good target function for comparison with the distribution of various cortical markers. In contrast, the deformation anisotropy (*α*) is set by the balance of forces acting on the cell wall and depends on the geometry of the cell end and its material properties.

### Measurement of material properties

The elastic properties of the fission yeast cell wall were measured using plasmolysis experiments. Cells coated with Qdots were deposited in a MatTek chamber containing 1 ml of YES medium. Drops of YES+2M sorbitol were added each 10 s while filming until the cells stopped diminishing in size (Supplementary Movie 4). The elastic properties were inferred using two complementary approaches. First, we focused on the change in geometry in the cylindrical mid-section of the cell. The meridional strains were evaluated by selecting pairs of Qdots on opposite ends of the mid-section and measuring the distance between them in the turgid and plasmolysed states. This approach yielded an average meridional strain of 0.13±0.005 (*n*=38). An average circumferential strain of 0.24±0.005 (*n*=38) was determined by comparing the width of the plasmolysed and turgid cells. The force balance in cylindrical pressure vessels dictates that the circumferential stress is twice the meridional stress and, accordingly, the Poisson’s ratio is given by the relation *ν*=(0.5*ρ*-1)/(*ρ*-0.5), where *ρ* is the ratio between the observed circumferential and meridional strains. For the value of *ρ* reported above, we found *ν*=−0.06 and *E/P*=44 (Fig. 2d). A second estimate of the elastic properties can be obtained by modelling the plasmolysis experiments using an axisymmetric shell model for the entire cell. Because of the important curvature observed in the plasmolysed state, it was necessary to include both bending and transverse shear terms to replicate accurately the experimental data (see Bending Model in the Supplementary Note). For simplicity, however, we assumed that the plasmolysed cell has no residual stresses and its material properties are homogeneous and isotropic. With these two assumptions, the only two material properties to determine were the ratio of the Young’s modulus over the turgor pressure *E/P* and the Poisson’s ratio *ν*. These material properties were estimated following three successive steps (Fig. 2d):

- The cell contours before and after plasmolysis were extracted manually by fitting a Bézier curve around the cells in bright-field images.
- The extracted contour was then made symmetrical.
- The contour corresponding to the plasmolysed cell was then numerically inflated using specific values of *E*/*P* and *ν*. The values yielding the best fit of the turgid state were retained. This approach gave values of *E*/*P*=58±15 and *ν*=0.033±0.005 (*n*=24). Therefore, our two approaches give a Poisson ratio very close to zero. Such low Poisson ratios have been predicted by Boal^58^ for 2D networks under high enough tension as could the case for the cell wall of *S. pombe*.

### Validation of assumptions

The quantification of cell morphogenesis from raw image sequences requires a number of assumptions. We regroup in this section the main assumptions used in our analysis and discuss how they were validated.

(i) Axisymmetry: An issue here is whether the morphogenesis of the cell ends is adequately captured by simply specifying the key variables along the meridian of the cell and assuming that all the variables are symmetric around the axis of the cell. This assumption would be incorrect if, for example, the cell ends curved substantially during growth. Axisymmetry is both a convenient simplifying assumption for computer simulations and an effective way to reduce noise in empirical measurements by allowing us to average measurements made on either sides of the cell axis. To test this assumption, we plotted the curvature and Qdot velocity for 19 OEs. A comparison of the values obtained to the left and right of the pole show that they are identical within the precision of our measurements (Supplementary Fig. 2). Moreover, both variables are properly fitted by functions where axisymmetry is forced. Finally, we note that axisymmetry would fail if the axis of growth were chosen incorrectly in our image analysis protocol. Therefore, the robust symmetry observed in Supplementary Fig. 2, in particular in the curvature profile, is also a validation of our protocol to selecting the pole of the cell.

(ii) Scalable steady-state assumption: Inspection of cell morphogenesis in *S. pombe* reveals somewhat unequal growth increments at the two cell ends. Much of the variation comes from an initial increase and final decrease in cell elongation associated with the period following and preceding mitosis^8^. However, even within the period of active end growth, variations in growth increments remain visible. These variations are often anticorrelated between the two ends of the cell. For example, relatively large growth increments at the OE are associated with relatively small growth increments at the NE^15^. These growth fluctuations have been attributed to temporal oscillations in cortical markers such as Cdc42 and its cofactors^15^.

We must conclude from these observations that fission yeast morphogenesis does not reach a steady state at any point during the cell cycle. In light of this, it would seem important to characterize the kinematic variables and marker profiles in terms of both their spatial and temporal variation. Yet, several lines of evidence underline the minor role played by the short-term and long-term fluctuations in shaping the cell ends.

1. We first tested whether the rise and fall of the growth rate during the cell cycle is important in determining cell shape. To do so, we exposed cells to HU to inhibit DNA replication. Under such conditions, growth is prolonged allowing OEs to elongate by 3.5 μm (*n*=7) on average, whereas the OEs of untreated cells elongate by only 2.0 μm (*n*=12). Despite the 75% increase in cell elongation, the curvature and meridional velocity of the HU-treated cells are indistinguishable from those of untreated cells (Supplementary Fig. 2a,b). We conclude from this experiment that OE geometry is not set by a precisely tuned rise and fall in growth rate.
2. If the short-term growth variations are important in controlling growth domain morphogenesis, they should make our measurements of Qdots velocities impossible to summarize with a single steady-state function. Indeed, our *raw* measurements of Qdot velocities show large fluctuations in magnitude (Supplementary Fig. 2d). However, the simple step of normalizing the Qdot displacements by the size of the growth increments is sufficient for the measurements to fall onto a unique master curve (Supplementary Fig. 2c). Thus, the growth variations affect only the magnitude of the Qdot velocities but not their meridional distribution.
3. As the growth fluctuations have been imputed to cycling of Cdc42 and its cofactors between the two cell ends, we tested whether the shape of the CRIB-3GFP fluorescence profile is affected by the overall intensity of CRIB-3GFP fluorescence (Supplementary Fig. 4b). To do so, we divided the frames of our CRIB-3GFP movies in two groups based on the total amount of apical fluorescence (the total fluorescence intensity integrated over the 12-μm-long curved contour spanning the pole). We observed that, after normalization, cell ends containing ‘high’ and ‘low’ levels of CRIB displayed nearly identical fluorescence profiles (Supplementary Fig. 4b).
4. Finally, we were unable to observe any repeatable pattern of fluctuation in all of the variables we quantified. In fact, the magnitude and temporal recurrence of the growth spurts are highly variable even in cells that otherwise show nearly identical morphogenesis. This variability excludes a direct one-to-one relationship between the growth spurts and the morphogenesis of the cell ends.

Taken together, these observations have led us to the idea of a ‘scalable’ steady state in which the spatial profile of time-dependent variables are preserved, although their absolute value may vary temporally as the growth of the cell end waxes and wanes.

(iii) Ergodicity of fluorescence profiles: We evaluated if ergodicity could be assumed for the distributions of cortical markers; that is, we checked that the canonical fluorescence profiles obtained by averaging over a population coincide with the profiles resulting from averaging single cells over time. For CRIB-3GFP, the existence of ergodicity was evidenced by filming cells expressing the marker (during 25 min every minute) and calculating the FWHA of the apical distribution profiles for each of the 25 time frames. Subsequently, we generated a histogram showing the frequencies of the FWHAs of single cells over those 2 h and another showing the population-based frequencies FWHAs (*n*=25 cell ends) and observed that they were similar (Supplementary Fig. 4a).

The low levels of intensity and the high rate of photobleaching of Sec6-GFP complicate tracking the distribution of that marker over long periods of time. Moreover, because of its uneven and dynamic distribution at the plasma membrane (Fig. 3b and Bendezú *et al*., 2011, where Sec6 is reported to have a half-time at the membrane of 5 s (ref. 30)), the information of single time-point frames—including the FWHA of the fluorescence profiles—is difficult to compare. However, we were able to film Sec6-GFP-expressing cells every minute during 30 min and observed that the average profiles were similar to the population average (*n*=29; Supplementary Fig. 4c).

The case of Bgs4 was slightly different, as that protein is very stable at the membrane, possibly because of its large N-terminal extracellular domains (see ref. 35 and Dodgson, Chessel *et al*., manuscript in preparation). When we filmed cells expressing RFP-Bgs4 we observed that the FWHA of the distribution of that protein barely changes over time or does so very gradually (Supplementary Fig. 4d; the separation between time points is 1 min). Moreover, as Supplementary Fig. 3b shows, the variability among the stable GFP-Bgs4 apical distributions is very low (the standard deviation of the FWHA is 0.19 μm; *n*=31). Based on those results, we concluded that the population average for Bgs4 reflects what happens cell-wise.

(iv) Fluorescence intensity is proportional to concentration: Many of the simulations we performed used as input what we call ‘canonical distribution profiles’, which resulted from averaging the apical distribution of the studied marker in: (i) a single cell over time, and/or (ii) many cells in the population in a steady-state context—for example, in growing OEs. For CRIB-3GFP, Sec6-GFP and RFP-Bgs4, which were taken as representative markers of the different components of the growth machineries, we carried out a battery of analyses to address how accurately the extracted fluorescence profiles reflect the behaviour of those markers during steady-state growth.

We checked for the saturation of the fluorescence intensity and saw no evidence for saturation for all of the markers. We assume that fluorescence intensity is proportional to concentration (and activity, as assumed in many other studies, for example, see ref. 59). We controlled for possible variations based on the type of fluorescent label (RFP versus GFP). As an internal control, we compared, using Prism (GraphPad Software Inc.), the GFP-Bgs4 with the RFP-Bgs4 data sets and found out that the differences between both are not statistically significant (*P*=0.71).

Owing to variations in the two radii of curvature of the cell end, the fluorescence intensities captured at specific meridional locations correspond to wall elements of slightly different surface areas. To correct for these variations, we computed the surface area associated with a small neighbourhood extending to a fixed distance, *h*, from the cell contour. The area of a small wall element is *A*=*dsdρ*, where *ds* is a small arc distance along the meridian and *dρ* is a small arc distance orthogonal to the meridian. Although *ds* is kept constant in our protocol to quantify the fluorescence, *dρ* is not. The length element *dρ* can be found based on the width *h* of the neighbourhood. We have *h*=*R*_*θ*_(1−cos*α*), where *R*_*θ*_ is the radius of curvature orthogonal to the meridian. Approximating cos*α* ∼1−*α*^2^/2 and rearranging, we find *α*=(2*h*/*R*_*θ*_)^1/2^. The length element is thus *dρ*=*R*_*θ*_*α*=(2*hR*_*θ*_)^1/2^ and the area is: *A*=*dsdρ*=*ds*(2*hR*_*θ*_)^1/2^. As *R*_*θ*_ varies only by about 10% and it enters as the square root, we expect little curvature effect on the recorded fluorescence. This conclusion was confirmed experimentally by quantifying the fluorescence distribution of the marker CaaX-GFP, which is targeted evenly to the plasma membrane^45^ (Supplementary Fig. 3a).

### Comparison of fluorescence profiles and morphogenesis

As shown in Fig. 2i,j, simulations of growth domain morphogenesis based on the measured areal expansion reproduce precisely the observed OE and NE morphogenesis. In light of these results, we hypothesized that markers whose apical distribution emulates closely the distribution of areal expansion—for example, polarity factors, the exocytic machinery and the cell wall regulating factors, all previously implicated in contributing to apical geometry in a variety of systems^25^,^26^,^30^,^60^,^61^,^62^,^63^—would be prime candidates for regulators of morphogenesis. To test this hypothesis, we used two metrics to assess the ability of markers to predict growth domain morphogenesis. The first comparison was based on the FWHA and FW95A of the profiles observed for the different markers and for the areal expansion (Supplementary Fig. 3c). The second metric looked directly at the predicted cell end geometries (specifically, the cell end curvature) for the various markers and compared them between themselves and against the observed cell end geometry (Supplementary Fig. 3d). We then used the Matlab functions pdist (euclidean distance) and linkage to cluster the various markers in terms of affinity. Although the topology and branch lengths of the clustering showed some slight variations depending on the metric used (Supplementary Fig. 3e), a robust consensus tree emerged from the analysis (Fig. 3c):

- Five markers cluster reliably closest to the areal profile. These are Tea1, the exocytic markers Sec6, Exo70 and Syb1, and the actin cable nucleator For3. Both Sec6 and Tea1 have a slightly narrower distribution than the observed areal expansion. Accordingly, simulations of OE morphogenesis using these markers yield slightly narrower cells but with nearly identical shape (Fig. 3c). However, the results regarding to Tea1 should be taken with caution, as the mean distribution of Tea1 might not be ergodic (the standard deviation is high; see Supplementary Fig. 3a,b; Tea1 is localized at the apical dome in discrete clusters with reduced motility^45^). Simulations using Syb1, Exo70 and For3 were able to reproduce well the shape of the canonical OE, although they resulted in slightly wider cells, maybe because the distribution of these markers have broad shoulders but short ‘tails’, unlike the bell shape geometry of the Areal profile.
- CRIB appears relatively isolated from the rest of the markers. The span of its fluorescence is comparable to the observed region of wall expansion but the shape of the distribution is such that the predicted OE shape is considerably more pointy than the observed canonical geometry (Fig. 3c).
- The glucan synthesis-related markers (Bgs1, Bgs4 and Rgf1) constitute a group that is placed significantly distant to the markers above. These markers show fluorescence tails extending far beyond the zone of active growth and cannot predict morphogenesis without first assuming some nonlinearity in the conversion of their fluorescence profiles into wall expansion.
- Finally, the Cdc42 GEF Scd1 and its cofactor Scd2 constitute the most distant group. These markers display a very narrow and pointy distribution. When used in our simulations, we obtain cells with implausibly narrow cell ends. These results and the distribution of CRIB described in (ii) (and assuming what Bendezú *et al*., 2015, have recently shown about the correlation of the localization of CRIB and Cdc42) indicate that the meridional distribution of Cdc42-GTP is not the result only of the localization of its activators but also of other factors (localization of other GEFs, GAPs, dynamics of Cdc42 and so on) that remain beyond the scope of this study^31^.

Taken together, these results suggest a special role for the secretion machinery in controlling growth domain morphogenesis.

### Distortion of the fluorescence profile

The fluorescence profiles (*γ*(*s*)) recorded in our experiments pertain to proteins involved at some level in specifying where wall assembly must take place. These proteins are positioned in the cortex and membrane of the cell where cytoskeletal dynamics and membrane recycling can control their lateral diffusion. In contrast, the breaking of load-bearing bonds in the wall and the insertion of new material must be effected by molecules residing in the periplasmic space and extracellular matrix. These molecules are likely to be outside the reach of the cellular processes able to confine cortical markers within a narrow distance of the pole. Consequently, the effectors of wall incorporation must flow with the wall in the same manner that Qdots flow with the expanding wall. The implication of this flow is a potential mismatch between the fluorescence profiles extracted for cortical markers and the actual process of wall incorporation taking place outside the cell.

To account for this potential mismatch, we are considering four physical processes. First, new wall material is deposited in the periplasmic space according to the experimentally recorded fluorescence profile (*γ*(*s*)). Second, the free wall material is incorporated in the load-bearing wall at a rate proportional to the local concentration of free polymer (*kχ*(*s*)). Third, the free wall material is spread out by the area expansion of the wall against which it lies according to the relation: *A*(*s*)*χ*(*s*). Finally, the wall is advected meridionally *v*(*s*)*∂χ*(*s*)/*∂s*. Note that we are not considering diffusion because the wall polymers are large molecules and are presumably entangle with the wall itself. The four physical processes lead to the following differential equation for the evolution of the free polymer concentration:




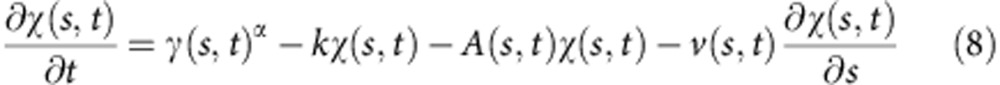




Here *χ* is the predicted, advected form of the fluorescence profile *γ* recorded in the experiments. Because we have measured the velocity of wall elements with Qdots, the only degrees of liberty are the two parameters *α* and *k*. They represent, respectively, a nonlinearity coefficient for the rate of deposition and the rate of incorporation of new wall material in the load-bearing matrix. According to the steady-state hypothesis, we will consider the steady-state solution of this equation, which means that the left-hand term is equal to zero. The steady-state advected distribution is:




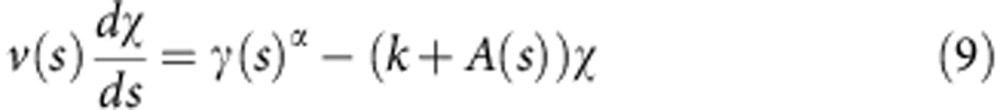




Substituting the explicit form for the areal strain, the solution to this equation is:




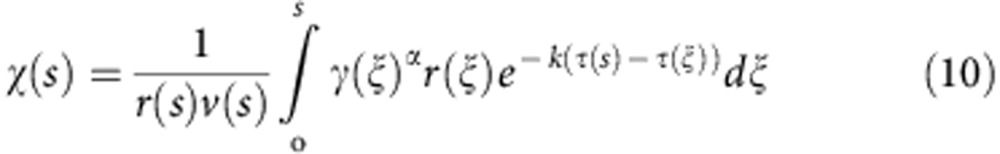




where *ξ* is a dummy variable of integration. The time of transit to point *τ*(*s*) is given by the integral:




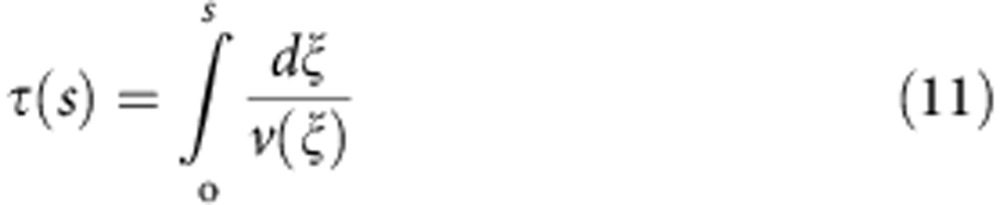




When *k* goes to infinity, the exponential approaches zero over the entire domain of integration except at *ξ*=*s* where it is equal to 1 as well as the functions that precede the exponential. Thus, for large *k*, the advected profile is close to the observed fluorescence profile. As *k* decreases with respect to the time of transit, the fluorescence becomes more ‘smeared’ laterally.

## Additional information

**How to cite this article:** Abenza, J. F. *et al*. Wall mechanics and exocytosis define the shape of growth domains in fission yeast. *Nat. Commun.* 6:8400 doi: 10.1038/ncomms9400 (2015).

## Supplementary Material

Supplementary Information Supplementary Figures 1-8, Supplementary Table 1, Supplementary Note 1 and Supplementary References

Supplementary Movie 1 Movie showing the changes in OE and NE shape in a fission yeast cell throughout interphase over 180 minutes. The frames, taken every 2 minutes, are Maximal Intensity Projections of 5.4 μm z-stacks. Bar, 5 μm

Supplementary Movie 2 Time-lapse movie of a growing Qdot-labelled cell over 120 minutes. Images were taken every 5 minutes using Optical Axis Integration (OAI) of the two most equatorial microns of the cell body. Left, Qdots; right, transmitted light. Bar, 5 μm.

Supplementary Movie 3 Time-lapse movie of a growing Qdot-labelled cell expressing GFP-Bgs4 imaged over 200 minutes. Left, Qdots; middle, GFP-Bgs4; right, transmitted light. Images were taken every 8 minutes using Optical Axis Integration (OAI) of the two most equatorial microns of the cell body.

Supplementary Movie 4 Time-lapse movie showing Qdot-labelled cells undergoing plasmolysis after the addition of sorbitol to the imaging medium, until a concentration of 1M was reached. The time separation between frames is 10 seconds. Bar, 5 μm.

Supplementary Movie 5 Simulation of cell morphogenesis in S. pombe based on wall elasticity and the pattern of exocytosis, here using specifically as a proxy Sec6-driven secretion. The colourmap highlights zones of high (red) and low (dark blue) Sec6 fluorescence.
