# Supplementary material for: Wall mechanics and exocytosis define the shape of growth domains in fission yeast
Source: Nat Commun. 2015 Oct 12;6:8400. doi: 10.1038/ncomms9400 (PMC4618311; doi:10.1038/ncomms9400)
Supplement: Supplementary Information — Supplementary Figures 1-8, Supplementary Table 1, Supplementary Note 1 and Supplementary References [file ncomms9400-s1.pdf]

Supplementary Figure 1: Kinematic analysis of fission yeast morphogenesis.

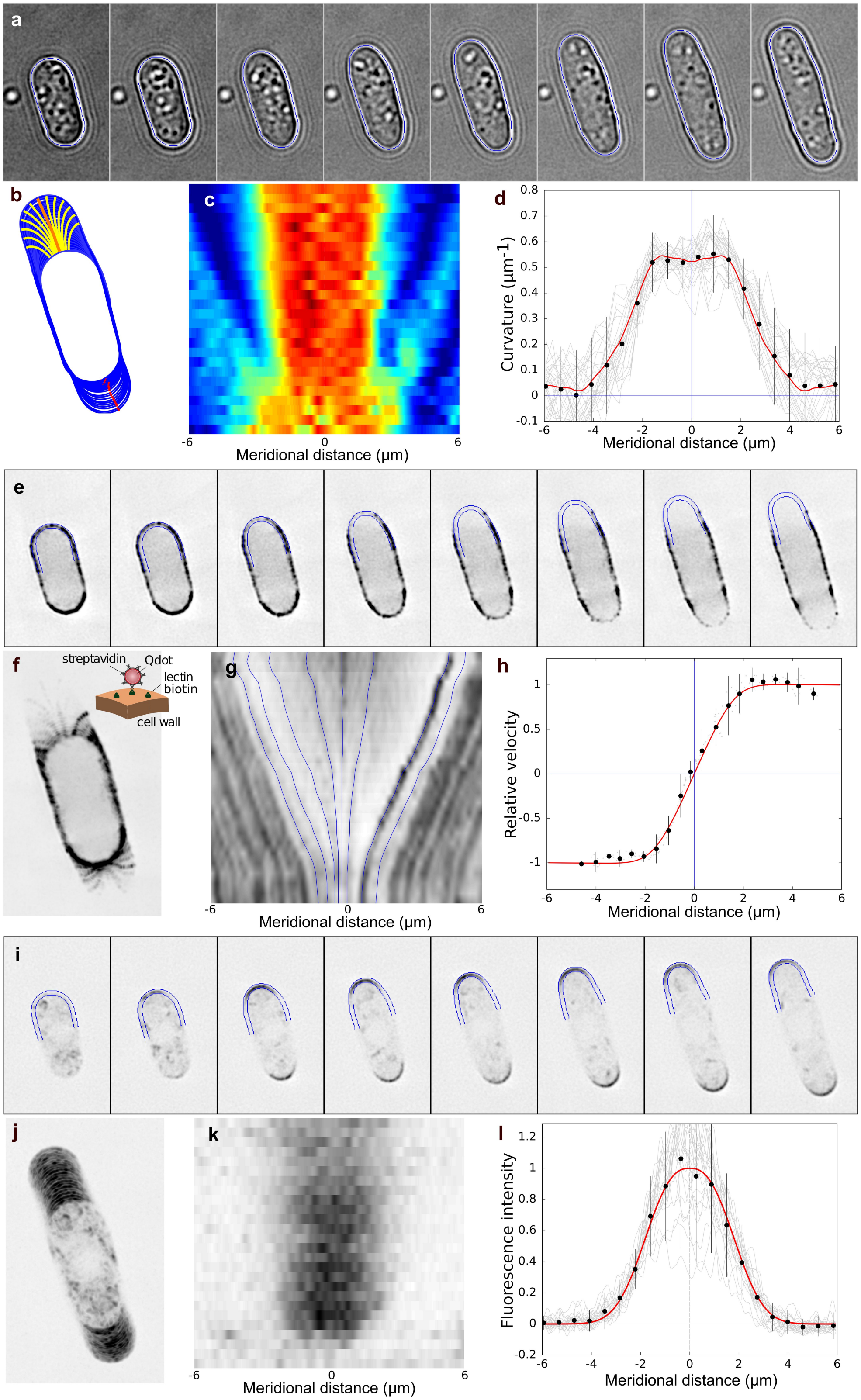

(a) Successive frames showing the tracking of the cell outline. **(b)** Overlaid cell outlines and orthogonal paths, including the growth axis (orange curve). **(c)** Kymograph of the meridional curvature of the OE. Time proceeds from bottom to top. The color scale ranges from dark blue (no curvature) to dark red (high curvature). **(d)** Meridional curvature for every time point (grey) and average of the curvatures computed over the entire growth sequence shown as mean and two standard deviations. The symmetrical temporal average of the curvature is shown as a red curve. **(e)** Successive frames showing a time-lapse image sequence of a cell decorated with Qdots and their tracking. **(f)** Maximum intensity projection of the Qdots' time-lapse sequence in **(e)**. The Qdots (emission wavelength, 605 nm), bound covalently to streptavidin, get attached to the cell wall of exponentially-growing cells after the intermediate addition of biotinylated isolectin. **(g)** Kymograph of Qdots fluorescence overlaid with their path. **(h)** Relative velocity of the Qdots with their average and two standard deviations. The raw velocity measurements were fitted with the symmetrical function $\upsilon(\varphi) = \sin\varphi(a + b\varphi^2 + c\varphi^4)$ (red curve) **(i)** Successive frames from a time-lapse image sequence showing the tracking of the fluorescence of a marker (here RFP-Bgs4). **(j)** Maximum intensity projection of the RFP-Bgs4 fluorescence in **(i)**. **(k)** Kymograph of the RFP-Bgs4 fluorescence. **(l)** RFP-Bgs4 fluorescence for individual frames with their average and two standard deviations. The raw fluorescence profile was fitted with a symmetric smoothing spline (red curve).

Supplementary Figure 2: Test of the axisymmetry and scaled steady-state assumptions.

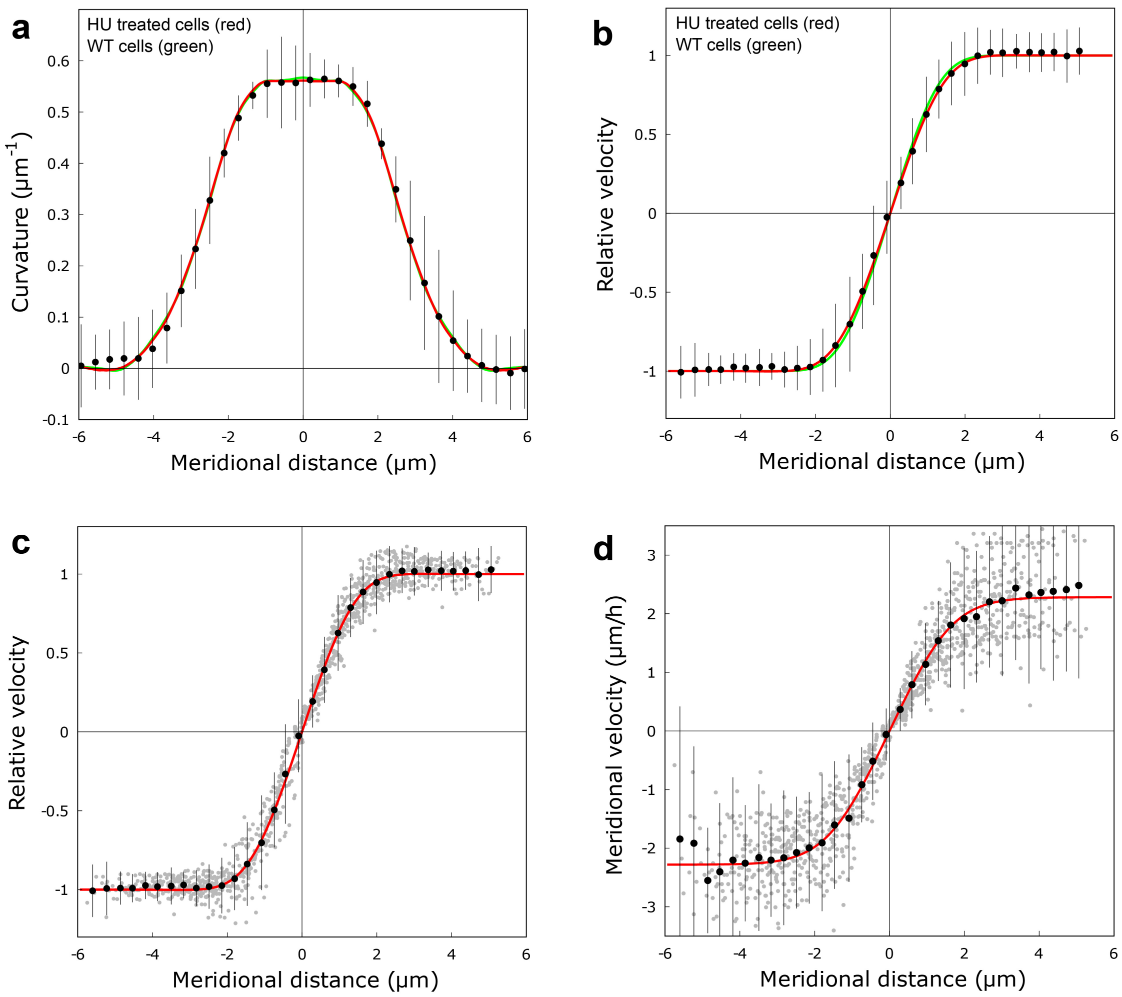

(a) and **(b)** Old end curvature and relative velocity for HU-treated cells (red curve, 7 cells) and untreated cells (green curve, 12 cells). Note that the two sides of the profiles were computed independently and overlaid with a symmetric best fit curve. The fits are equally good on either side of the cell confirming the validity of our axisymmetry assumption. **(c)** and **(d)** Meridional velocity computed with and without scaling based on the cell end advance. The scaled displacement shown in **(c)** allows the Qdot displacements to fall on the same master curve.

Supplementary Figure 3: Distribution of different key factors at the OE.

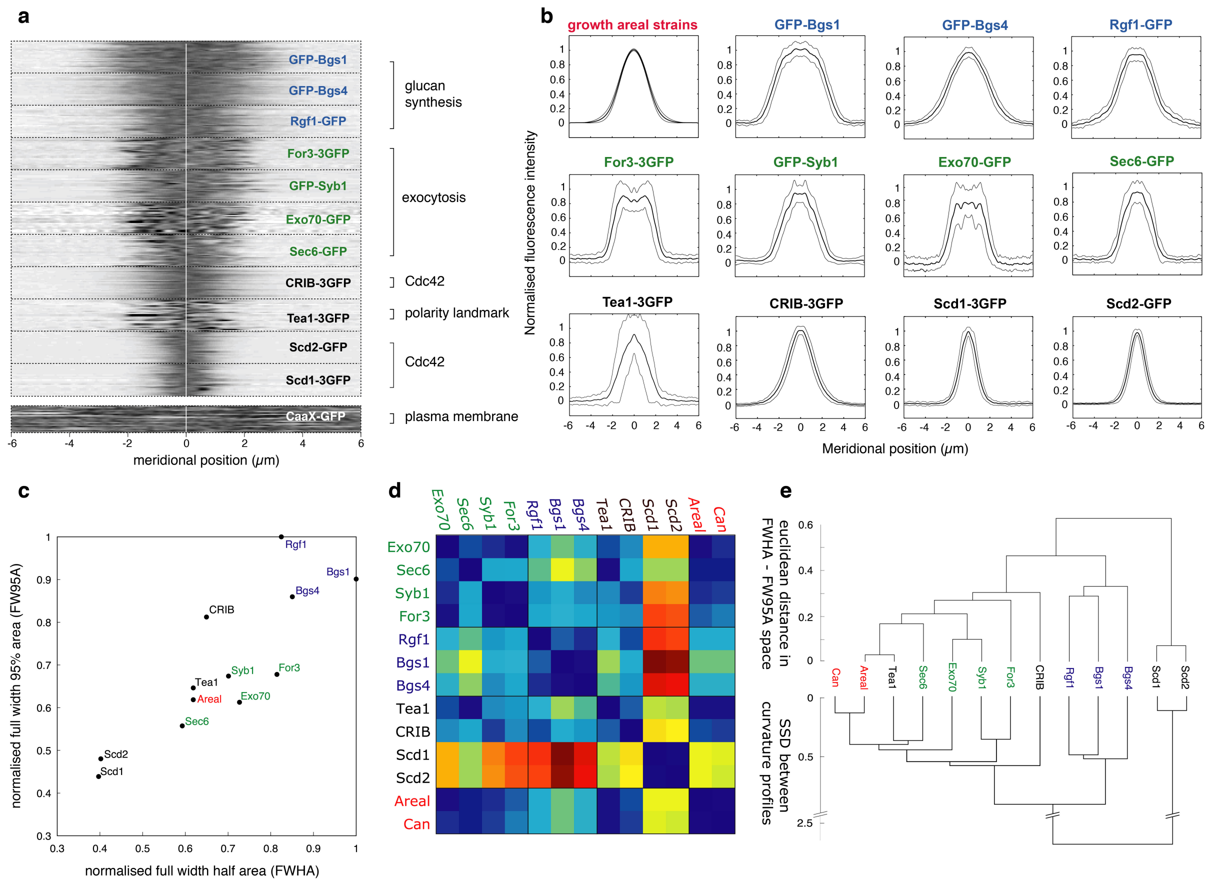

(a) Kymograph-like representation of the cortical distribution of the eleven GFP-labelled factors in n=29 OEs/factor. Images used were processed as in Figure 3b. The factors are grouped with regard to the specific pathway/machinery in which they theoretically take part. At the bottom of that plot, the distribution of the plasma membrane domain CaaX-GFP at 20 OEs is displayed in a semi-detached panel. **(b)** Plots showing the symmetrised average OE distribution of each GFP-labelled marker (thick line), obtained from n=29 OEs. The thinner lines demarcate the standard deviations. **(c)** Comparison of the fluorescence profiles and the measured areal expansion profile of the OE. The profiles are mapped according to their respective full width at half area (FWHA) and full width at 95% area (FW95A). **(d)** Colour map of the sum of squared differences (SSD) matrix obtained by comparing the observed

canonical curvature (Can), the predicted curvature for the measured areal expansion profile (Areal), and the predicted curvatures for each of the markers. The deep blue colour corresponds to a SSD of zero (i.e. the curvatures are identical) while a dark red colour corresponds to the highest SSD measured. **(e)** Comparison of the two dendrograms inferred from the analyses of the fluorescence profiles and predicted cell end curvatures. Note that the two approaches lead to nearly identical ranking of the molecular markers with only some minor changes in the tree topology and relative branch lengths. In particular, the secretion markers (Sec6, Exo70, Syb1) and the formin For3 form a well-defined group, which falls reliably closer to the observed profile of wall expansion and cell end curvature than the glucan synthesis markers (Bgs1, Bgs4, and Rgf1).

Supplementary Figure 4: Ergodicity can be assumed for the distributions of CRIB, Sec6 and Bgs4.

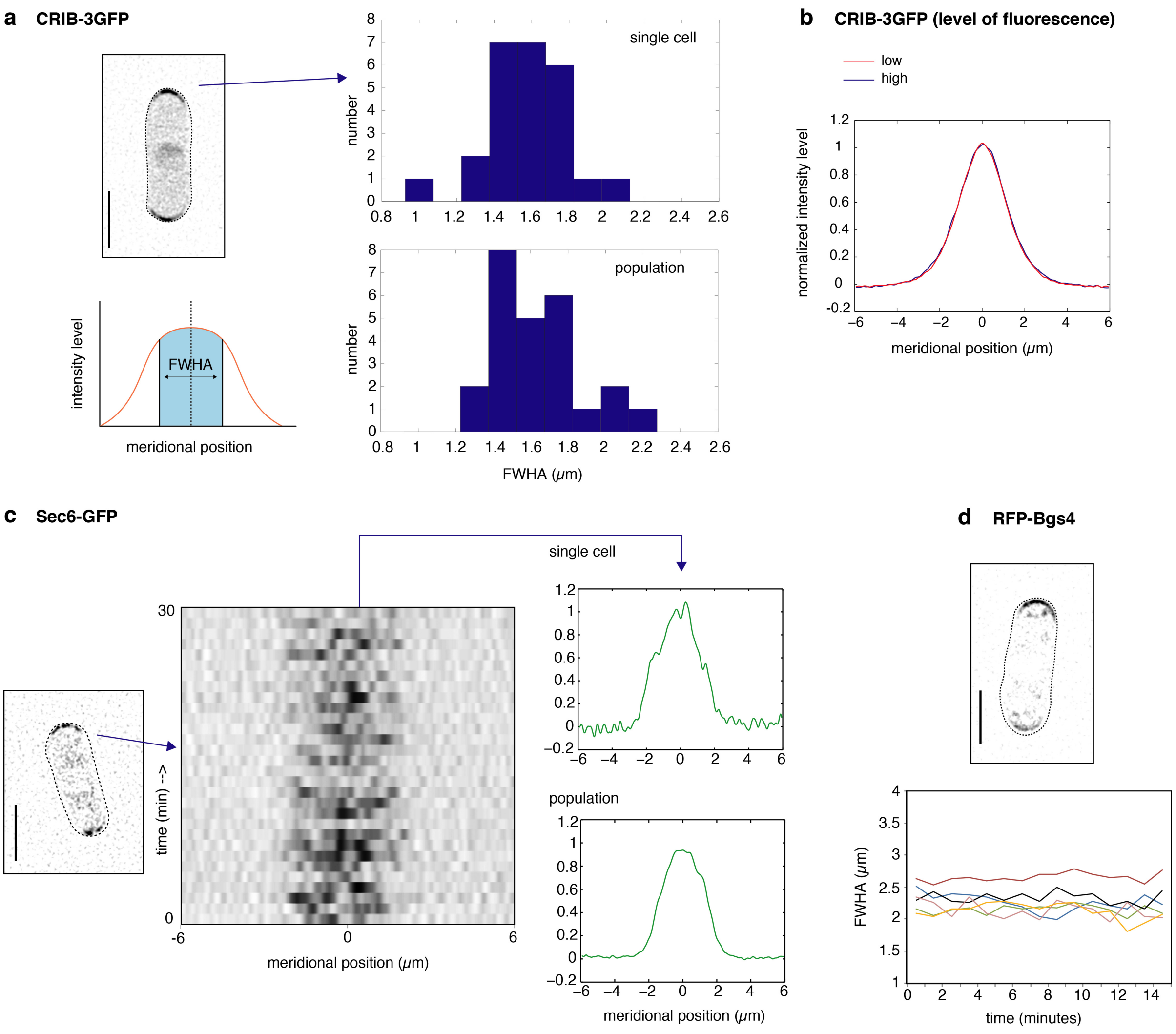

(a) Histograms showing, for the single cell displayed on the left, the number of time frames in which CRIB-3GFP exhibited a given range of FWHA (top; n=25) or, for a population (n=25), the number of cell ends expressing CRIB-3GFP in which the marker exhibited a given range of FWHA. **(b)** Plot showing that the normalised distribution of CRIB-3GFP at the cell ends is independent of its level of fluorescence (high: blue; low: red; see text) **(c)** Kymograph showing the apical distribution of Sec6-GFP in the OE of the cell depicted on the left over time (every minute during 30 minutes). On the right, the average profile of the

distribution of Sec6-GFP in the specified cell is compared with the distribution of that marker in a population of OEs (n=29; see Supplementary Fig. 3). **(d)** Plot representing the evolution of the FWHA of the distribution of RFP-Bgs4 in six different OEs (one of them belonging to the cell depicted on the left) over a period of 15 minutes.

Supplementary Figure 5: The choice of modelling parameters influences the similarity between predicted and actual cell end geometries

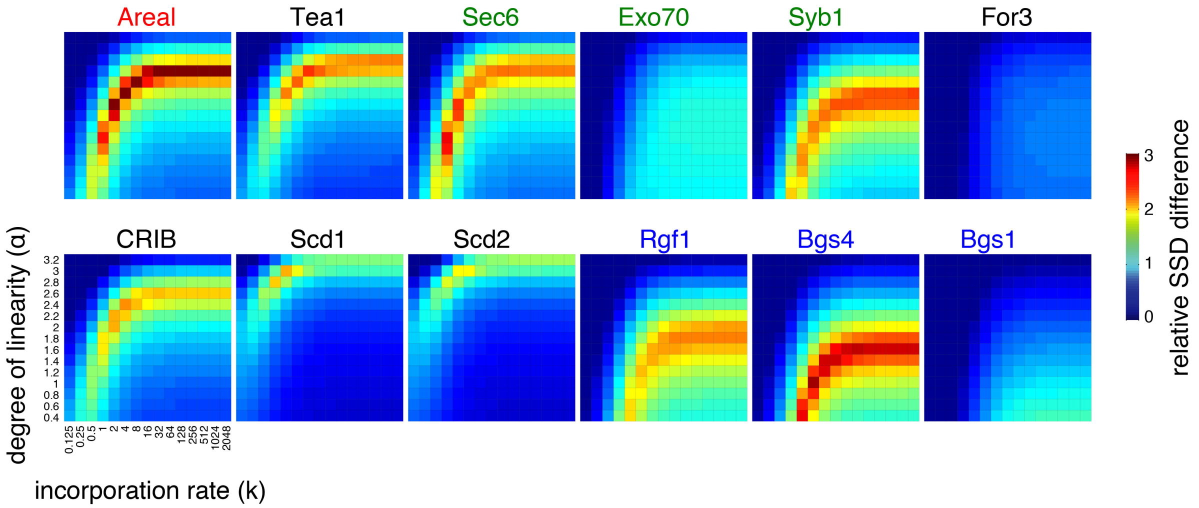

This "tile" representation shows the similarity of the geometry of a canonical OE (like the one represented in Fig. 1b) and a variety of *in silico* simulated OEs for a range of values of 'incorporation rate' (x axis; see Methods) and 'degree of linearity' (y axis; see Methods), using as a proxy the average fluorescence profiles of the eleven factors treated in our study. Specifically, the colours are based on the sum of squared differences (SSD) between the observed areal profile and the distorted fluorescence profile. Those SSDs have been then scaled by the maximal "possible" SSD (SSD_max). The log_10 of these was taken for the representation. The final equation is: log10(SSDmax/SSD). A value of 0 (dark blue) corresponds to a SSD equal to the SSD_max, while a value of 3 (dark red) corresponds to a SSD 1000 times less than the SSD_max, i.e. a very good fit of the areal profile.

Supplementary Figure 6: Distribution of the exocytic, glucan synthesis and Cdc42-related factors in wild-type, *rga2*Δ and *rga4*Δ.

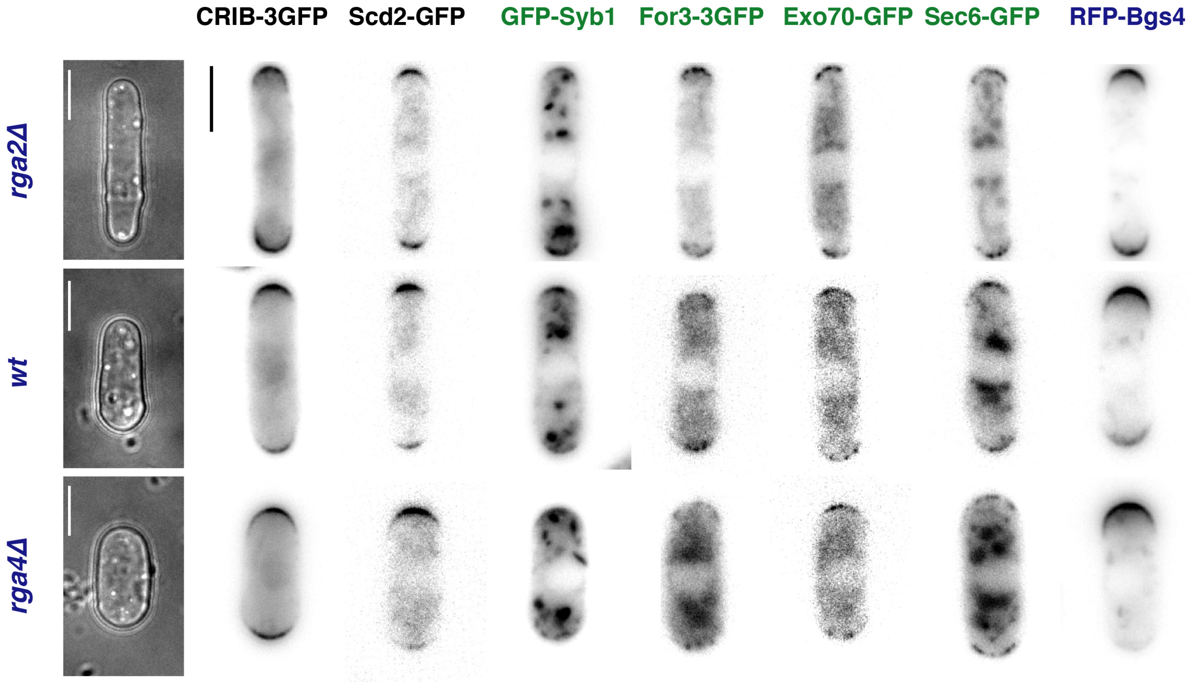

Images showing *rga2*Δ (top), wild-type (middle) and *rga4*Δ (bottom) cells expressing CRIB-3GFP, Scd2-GFP, GFP-Syb1, For3-3GFP, Exo70-GFP, Sec6-GFP and RFP-Bgs4. Bars, 5 μm. Transmitted light images of the mutants and wild-type are depicted on the left.

Supplementary Figure 7: The strong correlation between OE shape and exocytosis pattern remains in absence of actin cables.

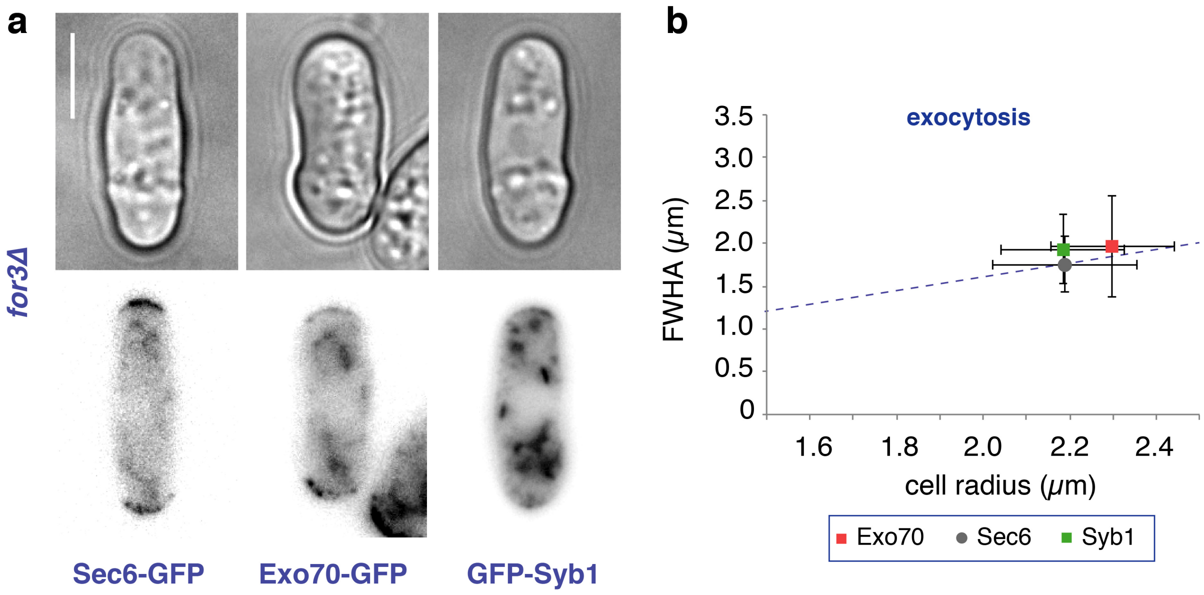

(a) Images showing the distribution of the exocyst subunits Sec6-GFP and Exo70-GFP and the v-SNARE GFP-Syb1 in a *for3*Δ background. Bar, 5 µm. **(b)** Plots illustrating the average FWHA of the distribution of those exocytic markers in the mutant *for3*Δ. n=30 cells/condition. The standard deviations are represented as crosses emerging from each average value (coloured shapes). The dashed line corresponds to the hypothetical ratio between the FWHA of the wild-type cell wall expansion strains and cell radius.

Supplementary Figure 8: The pattern of exocytosis at the new end prior to NETO might drive its evolution to OE.

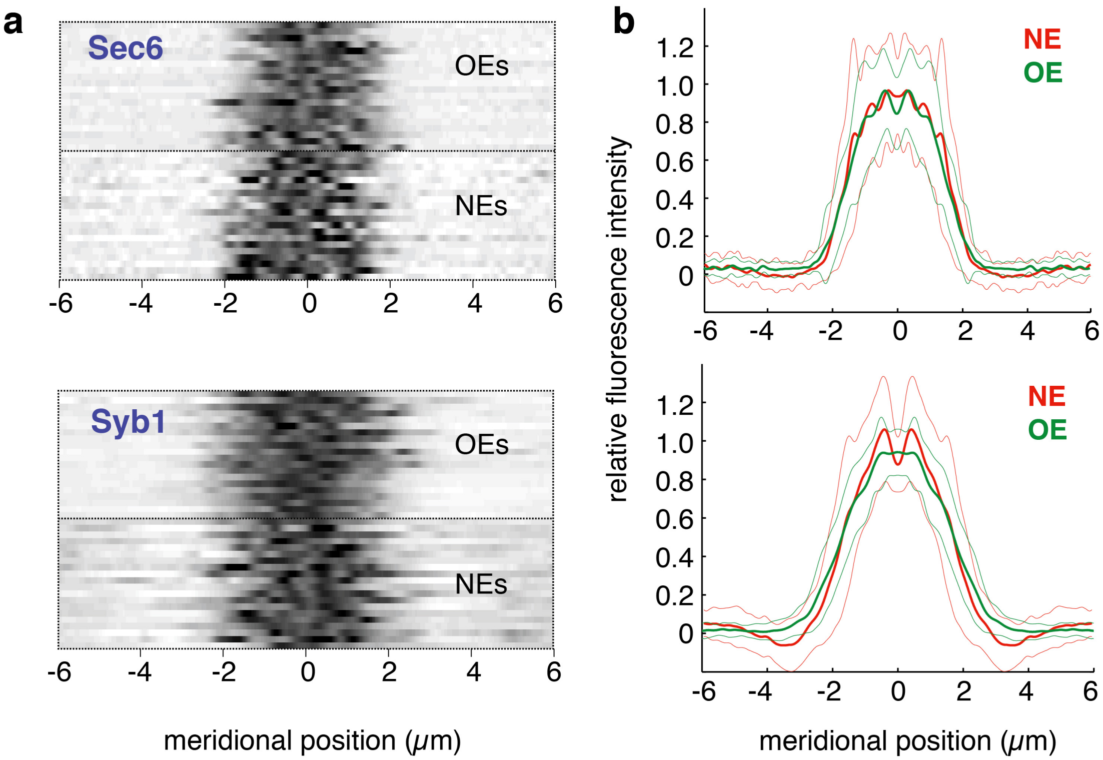

(a) Cortical distributions of Sec6-GFP and GFP-Syb1 in 20 OEs (top) and 20 pre-NETO NEs (bottom) *in vivo* (all the single-cell apical profiles are in inverted contrast and represented in single lines at the kymographs); **(b)** Average and standard deviations of those distributions. Their high similarity indicates that maintenance of OE geometry and evolution of the NE shape from hemispherical to OE-like are driven by cortical localization of the exocytic machinery.

Supplementary Table 1. Strains used in this study.

Strain	Genotype	Source
CA5931	*h- CRIB-3GFP::ura4*	Kaz Shiozaki
JCR519	*leu1-32 ura4-Δ18 his3-Δ1 bgs1Δ::ura4+ Pbgs1+::GFP-bgs1+:leu1+ h-*	[2]
JCR561	*leu1-32 ura4-Δ18 his3-Δ1 bgs4 Δ::ura4 Pbgs4-GFP-bgs4:leu1+ h-*	[3]
JFA190	*rgf1Δ::his3 his3Δ1? leu1-32? ade6M210? ura4Δ-18? leu1: rgf1-GFP rgf1-GFP:nat RFP-bgs4:hph*	This study
JFA194	*h- sec6-GFP:ura4 co2::Pshk1-CRIB-L-3mch:hph leu1-32 ade?*	This study
JFA204	*pal1Δ::kanMX6 CRIB-3GFP:ura4 ade-M210? leu1-32? ura4-Δ18?*	This study
JFA206	*pal1Δ::kanMX6 sec6-GFP:ura4 co2::Pshk1-CRIB-L-3mch:hph leu1-32 adeM-210?*	This study
JFA228	*CRIB-3GFP:ura4 Pbgs4:RFP-bgs4:leu1 bgs4Δ::ura4? rga4Δ::kanMX6*	This study

JFA230	*sec6-GFP:ura4 co2::Pshk1-CRIB-L-3mch:hph leu1-32 ade-M210? rga4Δ::kanMX6*	This study
JFA234	*CRIB-3GFP:ura4 Pbgs4:RFP-bgs4:leu1 bgs4Δ::ura4? rga2Δ::kanMX6*	This study
JFA235	*sec6-GFP:ura4 co2::Pshk1-CRIB-L-3mch:hph leu1-32 ade-M210? rga2Δ::kanMX6*	This study
JFA276	*h- rga2Δ::hph ade6-M210 leu1-32 ura4-Δ18*	This study
JFA309	*CO2::Padh1-GBP-mCherry-CaaX-hph leu1-32 ura4-Δ18? ade6-M216 (or ade 6-M210) GFP-Syb1-KanMX6*	This study
JFA312	*rga2Δ::hph Pbgs4^{+}-RFP-bgs4^{+}:leu1 bgs4Δ::ura4 for3-3GFP-kanMX6 ade6-M210? ura4-Δ18?*	This study
JFA314	*rga4Δ::hph exo70-GFP-KanMX6 ade6-M210 leu1-32 ura4-Δ18*	This study
JFA315	*rga4Δ::hph GFP-Syb1-kanMX6 ade6-M210? ura4-Δ18? leu1-32?*	This study
JFA316	*rga4Δ::hph Pbgs4^{+}-RFP-bgs4^{+}:leu1 bgs4Δ::ura4 for3-3GFP-kanMX6 ade6-M210?*	This study
JFA317	*rga2Δ::hph exo70-GFP-KanMX6 ade6-M210 leu1-32 ura4-Δ18*	This study
JFA319	*h+ for3Δ::hph ade-M210 leu1-32 ura4-Δ18*	This study
JFA322	*for3Δ::hph GFP-syb1-KanMX6 leu1-32 ade6-M210 (or ade6-*	This study

	M216) ura4Δ-14?	
JFA323	*for3Δ::hph exo70-GFP-KanMX6 ade6-M210 leu1-32 ura4-Δ18*	This study
JFA326	*for3Δ::hph sec6-GFP-ura RFP-bgs4-leu? bgs4Δ-ura? ura4-Δ18? leu1-32?*	This study
JFA328	*rga2Δ::hph GFP-syb1-KanMX6 ade6-M210? ura4-Δ18? leu1-32?*	This study
JFA333	*rga4Δ::hph Scd2-GFP-nat leu1-32? ade6-M210? ura4-Δ18?*	This study
JFA334	*rga2Δ::hph scd2-GFP-nat CRIB-3mCherry:ura4 leu1-32? ade6-M210?*	This study
MH365	*h- his7-366 leu1-32 ura4-Δ18 ade6-M216 Scd2-GFP-nat*	Masamitsu Sato
MH653	*h+ CO2::Padh1-GBP-mCherry-CaaX-hph leu1-32 ura4-Δ18 ade6-M216*	Masamitsu Sato
MP23E05	*pal1Δ::G418 bgs4::ura4+Pbgs4-RFP-bgs4::hph*	This study
PG40	*h- rgf1::his3 his3Δ1 leu1-32 ade6M210 ura4Δ-18 leu1+: rgf1+-GFP*	[4]
PN557	*ade-M216 leu1-32 ura4-Δ18 h-*	Paul Nurse
RCS0663	*pal1Δ::kanMX6 ade-M210 leu1-32 ura4-Δ18 h+*	Bioneer*

RCS0669	*tea1-3GFP:nat RFP-bgs4:hph h-*	This study
RCS0749	*leu1-32 uraΔ18 bgs4Δ::ura4 Pbgs4-RFP-bgs4:leu1 for3-3GFP:kanMX6*	This study
RCS0750	*CRIB-3GFP:ura4 Pbgs4-RFP-bgs4:leu1 bgs4Δ::ura4?*	This study
RCS0765	*kanMX6:GFP-syb1 ade6-210? leu1-32 ura4-Δ18 his3-Δ1? bgs4Δ::ura4+ Pbgs4-RFP-bgs4:leu1*	This study
RCS0766	*sec6-GFP:ura4 leu1-32 ura4-Δ18 his3-Δ1? bgs4Δ::ura4+ Pbgs4-RFP-bgs4:leu1*	This study
RCS1022	*pal1Δ::G418 sec6-GFP-ura4 co2::Pshk1-CRIB-L-3mch-hph leu1-32 ade6-M216?*	This study
YSM1528	*h- sec6-GFP-ura4 leu1-32*	[5]
YSM2075	*h- exo70-GFP-kanMX6 ade6-M210 leu1-32 ura4-Δ18*	[6]
YSM947	*h+ scd1-3xGFP:kanMX6 ura4-D18 leu1-32 ade6-M216*	Sophie Martin[7]

* http://pombe.bioneer.com [1]

SUPPLEMENTARY NOTE

In this note, we present two models for the mechanics of wall expansion and cell morphogenesis. Wall expansion involves the deposition of new wall material and the mechanical deformation of the pre-existing wall fabric. While elastic deformations of 5% are common in many walled cells [8], most mechanical models of walled cell morphogenesis do not include their contribution explicitely. Such omission is understandable since small elastic strains, although they may be important in controlling growth and have been considered as such, can hardly alter the cell geometry directly. The clearest evidence for this conclusion comes from observing the shape of plasmolysed walled cells, which rarely differs significantly from the shape of their turgid counterparts. In contrast, plasmolysis of actively growing *S. pombe* cells reveals large elastic deformations often exceeding 25%. Moreover, the relaxation of these elastic strains by plasmolysis can have a drastic effect on cell shape (Figure 2e in the main text). Therefore, we were compelled to include wall elasticity in our models of growth domain morphogenesis. Our simulation approach follows closely the approach adopted by Dumais *et al.* [9] and Rojas *et al.* [10] with one significant modification, the elastic loading of wall elements is also included explicitly. To implement the elastic loading of wall elements, we follow the approach of Goriely and co-workers [11] and decompose cell morphogenesis in two surfaces evolving in parallel (Figure A). These are the observed turgid surface of the cell and the relaxed (plasmolysed) surface. Every material point S of the relaxed surface is associated with a point $s(S)$ on the turgid surface. Under normal growth conditions, the cell maintains its turgor pressure throughout the cell cycle. Consequently, the relaxed geometry is never "experienced" directly by the cell; it is merely a ghost geometry that evolves in parallel with the turgid geometry. All physiological functions are performed within the confine of the deformed cell surface. Yet, a complete account of cell morphogenesis must include this virtual relaxed geometry since it is this surface that records faithfully the history of wall assembly.

Simulation of Cell Morphogenesis: Membrane Model

We model cell wall expansion as the product of two co-occurring processes. First, the wall is growing due to the action of enzymes which can break load-bearing bonds in the glucan network and insert new glucan chains. This growth process affects directly the relaxed geometry of the cell since new material becomes integrated in the wall. Second, the wall can respond elastically to any change in the forces it experiences. This elastic effect is encapsulated by the mapping between the relaxed and turgid cell geometries. This decomposition can be applied directly to the meridional velocity of material points as visualized by the displacement of Qdots. We have: $v(s(S)) = ds(S)/dt = (ds(S)/dS)(dS/dt) = \lambda_s(S)V(S)$, where $V(S)$ captures the flow associated with the incorporation of new wall material in the relaxed geometry and λ_s is the elastic stretching of wall element when the relaxed geometry is deformed by turgor pressure.

Substituting $v(s(S)) = \lambda_s(S)V(S)$ in the equations for the strain rates in an axisym-

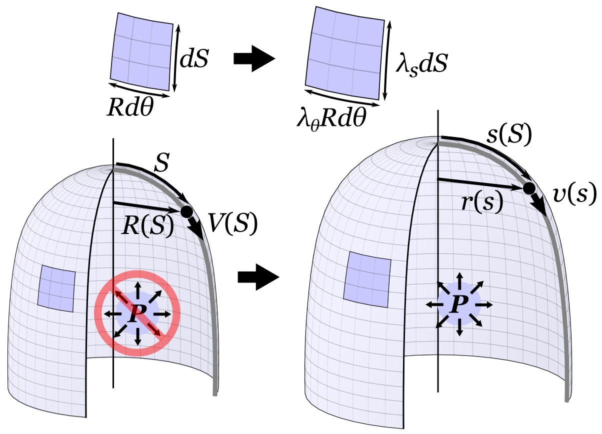

Figure A: Model of growth domain morphogenesis. The relaxed (left) and turgid (right) geometries of a growing fission yeast cell are illustrated. Growth by wall assembly is interpreted as an irreversible deformation of the relaxed cell geometry, $R(S)$. The severing of covalent bonds and insertion of new wall material induce a meridional flow of wall particles denoted $V(S)$. In the turgid cell indicated by the presence of turgor pressure P, the growth-induced flow field is distorted by the elastic stretching of the cell wall (λ_s and λ_θ), leading to a new flow field $v(s)$ reflecting both wall growth and wall elasticity.

metric shell, we get the following decomposition of the strain rates:

$$\dot{\epsilon}_s = \frac{dv}{ds} = \frac{d\lambda_s V}{ds} = \frac{1}{\lambda_s}\frac{d\lambda_s}{ds}v + \frac{dV}{dS} \tag{1}$$

$$\dot{\epsilon}_\theta = \frac{1}{r}\frac{dr}{ds}v = \frac{1}{\lambda_\theta}\frac{d\lambda_\theta}{ds}v + \frac{1}{R}\frac{dR}{dS}V \tag{2}$$

where the stretch ratios $\lambda_s = ds(S)/dS$ and $\lambda_\theta = r(s(S))/R(S)$ were used.

Simulation algorithm – We have implemented the model described above with a sequences of seven steps applied recursively.

i) We first input the observed curvature (i.e. the growth domain geometry) and fluorescence profile from a particular experiment. This step sets the initial conditions for the simulations and provides a benchmark to evaluate the ability of a given cortical marker to reproduce the morphogenesis of the OE and NE.

ii) We compute the elastic strains and stresses for the geometry. The membrane stresses are given by the force balance between the cell's turgor pressure (P) and the tensions in the wall surrounding the cell. Given that the wall is thin compared to the typical radius of the cell, the meridional and circumferential stresses can be expressed

directly in terms of the turgor pressure and the local geometry of the cell surface [9]:

$$\sigma_s = \frac{P}{2\delta\kappa_\theta} \tag{3}$$

$$\sigma_\theta = \frac{P}{2\delta\kappa_\theta}\left(2 - \frac{\kappa_s}{\kappa_\theta}\right) \tag{4}$$

where $\delta = 0.2\mu m$ is the wall thickness, and κ_s and κ_θ are the principal curvatures of the surface.

To compute the elastic strains, we used the stress relations above and the material properties ($\nu = 0.3$ and $E/P = 40$) which, within a broad domain of morphogenetically compatible material properties, were closest to the material properties measured in the plasmolysis experiments (Figure 2d in the main text). The equations for the elastic strains are:

$$\epsilon_s^e = \frac{1}{E}(\sigma_s - \nu\sigma_\theta) \tag{5}$$

$$\epsilon_\theta^e = \frac{1}{E}(\sigma_\theta - \nu\sigma_s) \tag{6}$$

For simplicity, these equations assume the cell wall to be isotropic, homogeneous and linearly elastic. It is likely that these assumptions are not perfectly satisfied although any more complex model would add free parameters which so far have not been measured.

iii) We compute the strain rates associated with growth. The growth strain rates are:

$$\dot{\epsilon}_s^g = \alpha\gamma(s)\epsilon_s^e \tag{7}$$

$$\dot{\epsilon}_\theta^g = \alpha\gamma(s)\epsilon_\theta^e \tag{8}$$

where $\gamma(s)$ is the fluorescence intensity profile of the marker of interest and α is a factor relating the fluorescence intensity with the activity of the marker in question. We note that this factor has no effect of the shape of the growing cell end but sets the rate of elongation.

iv) We compute the meridional velocity of material points. The meridional velocity is simply the spatial integral of the meridional strain rate $\dot{\epsilon}_s^g$ computed above.

v) We compute the rate of elastic loading due to the growth process. The rate of elastic loading is the second contribution to the deformation of wall elements. It is given by the relations: $\dot{\epsilon}_s^e = \frac{1}{\lambda_s}\frac{d\lambda_s}{ds}v$ and $\dot{\epsilon}_\theta^e = \frac{1}{\lambda_\theta}\frac{d\lambda_\theta}{ds}v$.

vi) We compute the Lagrangian velocity field from the total strain rates. The total strain rates are the sum the contribution of strain rates arising from growth of the undeformed cell geometry ($\dot{\epsilon}_s^g$ and $\dot{\epsilon}_\theta^g$) and elastic loading of wall elements ($\dot{\epsilon}_s^e$ and $\dot{\epsilon}_\theta^e$). For an axisymmetric cell, the two total strain rates can be integrated to give the normal and tangential speed of displacement of material points [9]. The integration gives:

$$v_t(s) = \sin\varphi(s)\int_S^s \frac{1}{\sin\varphi(s)}\left(\dot{\epsilon}_s - \frac{\kappa_s}{\kappa_\theta}\dot{\epsilon}_\theta\right) ds \tag{9}$$

$$v_n(s) = \frac{\dot{\epsilon}_\theta}{\kappa_\theta} - \cos\varphi(s) \int_S^s \frac{1}{\sin\varphi(s)} \left(\dot{\epsilon}_s - \frac{\kappa_s}{\kappa_\theta}\dot{\epsilon}_\theta \right) ds \tag{10}$$

vii) We take a small step forward to find the new geometry. Given the velocity of every point on the meridian, the new, deformed, geometry is determined by finding the position of each point after a small time interval. This process maps the material point trajectories as if the were Qdots. During a growth interval, some material points are displaced from the growing dome to the non-growing cylinder. These points are eliminated and replaced by new points within the growth region such that the length of the growth region and its spatial resolution are preserved. This is done by fitting the deformed meridian with a cubic spline and remeshing with a uniform spacing between points.

Steps *ii* to *vii* are repeated until a steady state is reached.

Validation - Since we are using the growth simulations to test the compatibility between the distribution of molecular markers and the geometry of the fission yeast cell, it is imperative that the simulation protocol be exact at the kinematic level; that is, all the geometrical relations embedded in the implementation of the model must be exact to any accuracy desired. We first confirmed the convergence of our algorithm when run with the analytical solution for the isotropic growth of a spherical cap (Figure B). According to this solution, the strain rates must vary as the cosinus of the angle of the surface normal. Second, we verified that the kinematics is "closed"; that is, it is possible to reproduce the geometry of a given cell exactly when the strain rates extracted for this cell are used as input to our simulation protocol (Figure B).

Simulation of Cell Morphogenesis: Bending Model

The membrane assumption, which states that turgor pressure is supported only by in-plane tensions in the wall, is widely employed to model the growth of walled cells such as fungal hyphae and pollen tubes. Its main attributes are its robustness and simplicity. The aim of the bending model is describing not only the growth of the cell end but the whole 'morphogenetic' cell cycle including the deformation of the septum into the NE following cell division. Because of the large change in curvature at the septum, the description of division must include both bending and transverse shear terms in the shell equation. Our bending model of yeast cell growth is based on the model of Su and Taber [12]. The equations were solved for an axisymmetric shell approximating the yeast cell geometry. However, as yeast cells grow without twist, we discarded all the terms in Su and Taber's equations which correspond to twist. The boundary conditions are that the two curvatures (κ_{11}, κ_{22}) and two deformations ($\lambda_{11}, \lambda_{22}$) are equal on the two points laying on the axis of rotational symmetry, while the transverse shear Q_2 is 0 at those points. We solved the governing equations according to the method outlined by Kempski *et al.* [13] and making use of the function fsolve in Matlab (see below).

While testing our model on an inflating sphere, we observed that the set of equations

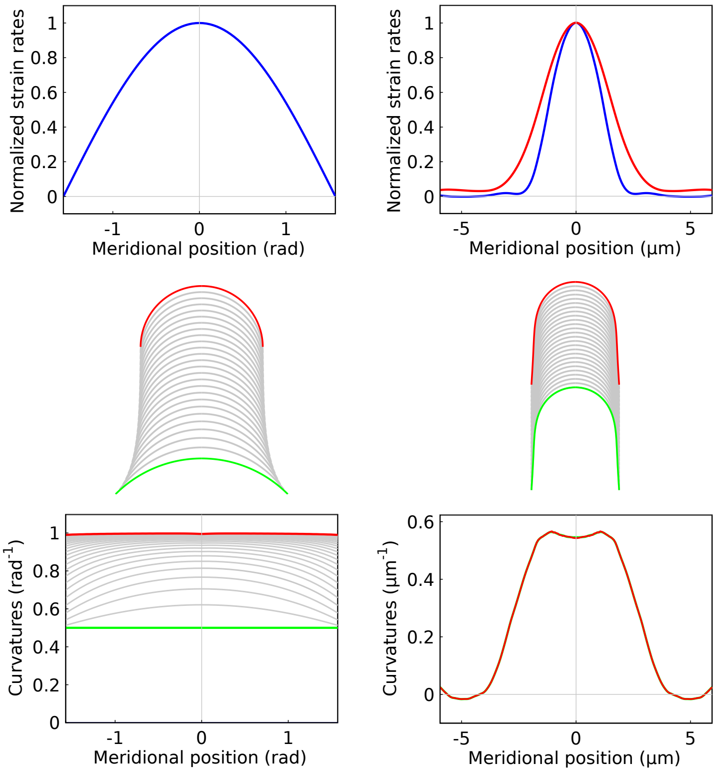

Figure B: Two simulations validating the kinematics of our implementation of the model. (Left) Growth domain morphogenesis using the analytical strain rates solution for an isotropically growing hemisphere. The cell end is seen to converge precisely to the specified hemisphere. (Right) A simulation testing the "closure" of the kinematic equation. In this simulation, the cell end is grown with the strain rates observed in a specific cell. The simulation propagates the cell end shape and curvature without any distortion indicating that the kinematics equations are exact.

proposed by Su and Taber does not maintain the spherical geometry even at quite low deformation. We found that this asymmetry originates from an oversimplification proposed by Reissner [14] using r_0 rather than r in the derivative of the ODE. After removing the simplification, the inflating sphere remained spherical. The system we ultimately used corresponds to the one proposed originally by Reissner (before the simplification) including the transverse shear term of Su and Taber.

The system of equations behind the bending model is:

$$\partial_s r = \lambda_{22}\cos(\phi) + \gamma_2 \sin(\phi) \tag{11}$$
$$\partial_s z = \lambda_{22}\sin(\phi) - \gamma_2 \cos(\phi) \tag{12}$$
$$\partial_s \phi = \alpha(\kappa_{22} + \partial_s \phi_0) \tag{13}$$
$$\partial_s V_{2b} = -r\alpha p_v \tag{14}$$
$$\partial_s H_{2b} = \alpha(\frac{\partial w}{\partial \lambda_{11}} - r p_h) \tag{15}$$
$$\partial_s M_{22b} = \alpha(\cos(\phi)\frac{\partial w}{\partial \kappa_{11}} + r Q_2 \lambda_{22} - r H_2 \gamma_2) \tag{16}$$

where the variables are as follow:
r, z are the radial and axial coordinates of the axisymmetric contour.
ii=11, 22 indicates the circumferential and meridional directions, respectively.
λ_{ii} is the deformation along direction ii.
γ_2 corresponds to the shear deformation.
κ_{ii} is the difference between rest state curvature and deformed state curvature along direction ii.
Γ_s corresponds to the shear angle.
N_{ii} are the stresses along direction ii.
N_{22} is decomposed between vertical component V_2 and horizontal component H_2.
Q_2 is the transverse shear stress.
M_{ii} are the bending moments in direction ii.
Finally, p_v and p_h are the vertical and horizontal components of the turgor pressure, w is the elastic energy, E is the Young's modulus, and ν is the Poisson's ratio.

Other parameters are used for numerical purposes $(H_{2b}, V_{2b}, M_{22b}, \alpha)$. They are defined by the following relations:

$$H_2 = \frac{H_{2b}}{r},\ M_{22} = \frac{M_{22b}}{r},\ V_2 = \frac{V_{2b}}{r} \tag{17}$$
$$N_{22} = H_2\cos(\phi) + V_2\sin(\phi),\ Q_2 = H_2\sin(\phi) - V_2\cos(\phi) \tag{18}$$
$$\lambda_{11} = \frac{r}{r_0},\ \kappa_{11} = \frac{\sin(\phi)}{r} - \frac{\sin(\phi_0)}{r_0},\ \alpha = \sqrt{\gamma_2^2 + \lambda_{22}^2} \tag{19}$$
$$\Gamma_s = \arctan(\frac{\gamma_2}{\lambda_{22}}),\ p_h = p\sin(\phi - \Gamma_s),\ p_v = -p\cos(\phi - \Gamma_s) \tag{20}$$

The following linear elastic energy including both transverse shear and bending was sufficient to describe plasmolysis experiments.

$$\begin{aligned} w(\lambda_{11}, \lambda_{22}, \gamma_2, \kappa_{11}, \kappa_{22}) &= A((\lambda_{11}-1)^2 + 2\nu(\lambda_{22}-1)(\lambda_{11}-1)(\lambda_{22}-1)^2) \\ &+ B\gamma_2^2 + C(\kappa_{11}^2 + 2\nu\kappa_{11}\kappa_{22} + \kappa_{22}^2) \end{aligned} \tag{21}$$

where $A = \frac{Eh}{2(1-\nu^2)}$, $B = \frac{Eh}{4(1+\nu)}$, and $C = \frac{Eh^3}{24(1-\nu^2)}$. The system of ODE was closed by calculating the following variables λ_{22}, κ_{22}, γ_2 :

$$\lambda_{22} = \frac{(1 + \frac{(1-\nu^2)N_{22}}{Eh} - \nu(\lambda_{11}\lambda_{11}^* - 1))}{\lambda_{22}^*} \tag{22}$$

$$\gamma_2 = \frac{2Q_2}{Eh} \tag{23}$$

$$\kappa_{22} = \frac{12M_{22}}{Eh^3} - \nu\kappa_{11} \tag{24}$$

Simulation algorithm - For matter of clarity, the preceding system of equations is rewritten in the following form:

$$0 < \quad s \quad < l \tag{25}$$

$$u \quad = \quad (r, z, \varphi, V_{2b}, H_{2b}, M_{22b}) \tag{26}$$

$$\dot{u} \quad = \quad f(u, s, p) \tag{27}$$

$$0 \quad = \quad g(u, s, p) \tag{28}$$

f stands for the differential equations (eqns. 11-16). g stands for the algebraic equations (eqns. 22-24). l stands for the total arclength of the plasmolysed contour. Two functionals of u are introduced:

$$BC(u) \quad = \quad (\lambda_{11} - \lambda_{22}, \kappa_{11} - \kappa_{22}, Q_2) \tag{29}$$

$$F(u, s, p) \quad = \quad u(s) - u(0) - \int_0^s f(u, p) \tag{30}$$

Solving the boundary condition problem is equivalent to finding a function u which is a zero of F and g on the whole interval $]0, l[$ and a zero of BC at both poles.

To solve the equations, we first defined the initial solution U_0 using the plasmolysed, stress-free contour ($p_0 = 0$). Then the pressure is increased by a small step δ : $p_n = p_{n-1} + \delta$. The problem is discretised in the following way. Two new functionals G and H are defined:

$$G(U, s, p_n) \quad = \quad [F(U, s, p_n)g(U, s, p_n)] \tag{31}$$

$$H(U, p_n) \quad = \quad ([G(U, s_1, p_n) \ldots G(U, s_i, p_n) \ldots G(U, s_N, p_n)], BC(U, 0), BC(U, l)) \tag{32}$$

The problem $H(U, p_n) = 0$ is solved using fsolve of Matlab among the cubic splines whose nodes are linearly spaced between 0 and the arclength l of the initial contour. As f diverges at both poles, the function G inside the function H is evaluated at the s_i which

are evenly distributed between $(\epsilon, l-\epsilon)(\epsilon = 10^{-5})$. The s_i are distributed on the whole interval but are denser close to the pole. The guess function used to initiate the function fsolve is the cubic spline U_{n-1}. This numerical code was validated by calculating the solutions for geometries where the boundary condition problem admits analytical solutions for the whole contour (the sphere) or for part of the contour (a very elongated cylinder with two hemispherical cell ends). For these geometries both analytical and numerical solutions match very well.

In order to model the transition between NE and OE, a growth model including bending terms was implemented. In this second model the strains induced by the turgor are directly calculated from the plasmolysed state whereas they were evaluated from the deformed configuration in the first model. The set of equations used to describe growth conserves the ODE system (eqns. 11-16) but each of the strains and the bending deformations are decomposed in two subvariables:

$$\lambda_{ii,n} = \lambda_{ii,n}^{step} \lambda_{ii,n}^{residual} \tag{33}$$

$$\kappa_{ii,n} = \kappa_{ii,n}^{step} \kappa_{ii,n}^{residual} \tag{34}$$

The "residual" part is determined at the precedent growth step. The "step" part is calculated at the current step. The system of ODE (eqns. 11-16) is now closed by the seven following algebraic equations:

$$\lambda_{22}^{step} = \frac{1 + \frac{N_{22}}{2A} - \nu(\lambda_{11} - 1)}{\lambda_{22,n}^{residual}} \tag{35}$$

$$\gamma_2 = \frac{Q_2}{2B} \tag{36}$$

$$\kappa_{22}^{step} = \frac{M_{22}}{2C} - \nu(\kappa_{11}) - \kappa_{22,n}^{residual} \tag{37}$$

$$\lambda_{11} = \lambda_{11}^{step} \lambda_{11,n}^{residual} \tag{38}$$

$$\lambda_{22} = \lambda_{22}^{step} \lambda_{22,n}^{residual} \tag{39}$$

$$\kappa_{11} = \kappa_{11}^{step} + \kappa_{11,n}^{residual} \tag{40}$$

$$\kappa_{22} = \kappa_{22}^{step} + \kappa_{22,n}^{residual} \tag{41}$$

The following steps were followed.

i) The initial conditions used for the simulations were the geometry of the plasmolysed cell with the material properties (Poisson's ratio $\nu = 0.033$ and a wall thickness $h = 0.2\mu\text{m}$). The initial geometry is assumed to be relaxed (free of bending and membrane stresses):

$$\lambda_{ii,0}^{residual} = 1 \tag{42}$$

$$\kappa_{ii,0}^{residual} = 0 \tag{43}$$

ii) The initial geometry subsequently swelled up to a ratio E/P of 58. The "step" part of the strains and bending strains equal the strains and bending strains induced by this swelling.

$$\lambda_{ii,0} = \lambda_{ii,0}^{step} \quad (44)$$
$$\kappa_{ii,0} = \kappa_{ii,0}^{step} \quad (45)$$

iii) The new curvilinear coordinate s_n is calculated:

$$s_n(s_{n-1}) = \int_0^{s_{n-1}} \lambda_{22,n-1}^{step} ds_{n-1} \quad (46)$$

iv) The residual strain and bending strain are defined:

$$\lambda_{ii,n}^{residual} = \frac{\lambda_{ii,n-1}}{1 + \delta K(\lambda_{ii,n-1} - 1)} \quad (47)$$
$$\kappa_{11,n}^{residual} = \frac{\kappa_{ii,n-1}}{1 + \delta K(\lambda_{ii,n-1} - 1)} \quad (48)$$

These prefactors correspond physically to an increase of the rest length proportional to the deformation $(\lambda_{ii,n-1} - 1)$ times the fluorescence profile K of the cortical marker we want to study. The proportionality factor δ quantifies the amplitude of each step of the growth.

v) The grown shape of cell is obtained by solving eqn. 32 with fsolve where the function g inside the formula (eqn. 31) is replaced by the condition (eqns. 35-41). The guess function used to initiate fsolve is:

$$U_n^{guess} = (r_{n-1}, z_{n-1}, \varphi_{n-1}, V_{2b,n-1}, H_{22b,n-1}, \lambda_{11}^{step} = 1, \kappa_{11}^{step} = 1, \kappa_{22}^{step} = 1, \gamma_2) \quad (49)$$

The last three steps are reiterated until an asymptotic cell end shape is reached. Provided that δ is small enough (< 0.3), its values does not affect the asymptotic shape. We in fact found that the simulations converge to the same asymptotic cell end geometry irrespective of the initial conditions (Figure C).

The way of including the bending in the growth of shell is still a matter of debate. For this reason we adopted the simplest approach with no term of bending in the growth but a term of bending in the energy. As a control of robustness of the hypothesis, we carried out two supplementaries sets of simulations one with no term of bending in the energy and another one with the most naive way of including the growth in the bending strain (eqn. 48) by multiplying the rest radius of curvature by the same factor as the rest length in (eqn. 47). The first method did not affect the attractor at all for both the asymptotic curvature and the asymptotic diameter but it changes the dynamic of the curvature because of the initial bending momentum. The second method gives a slightly

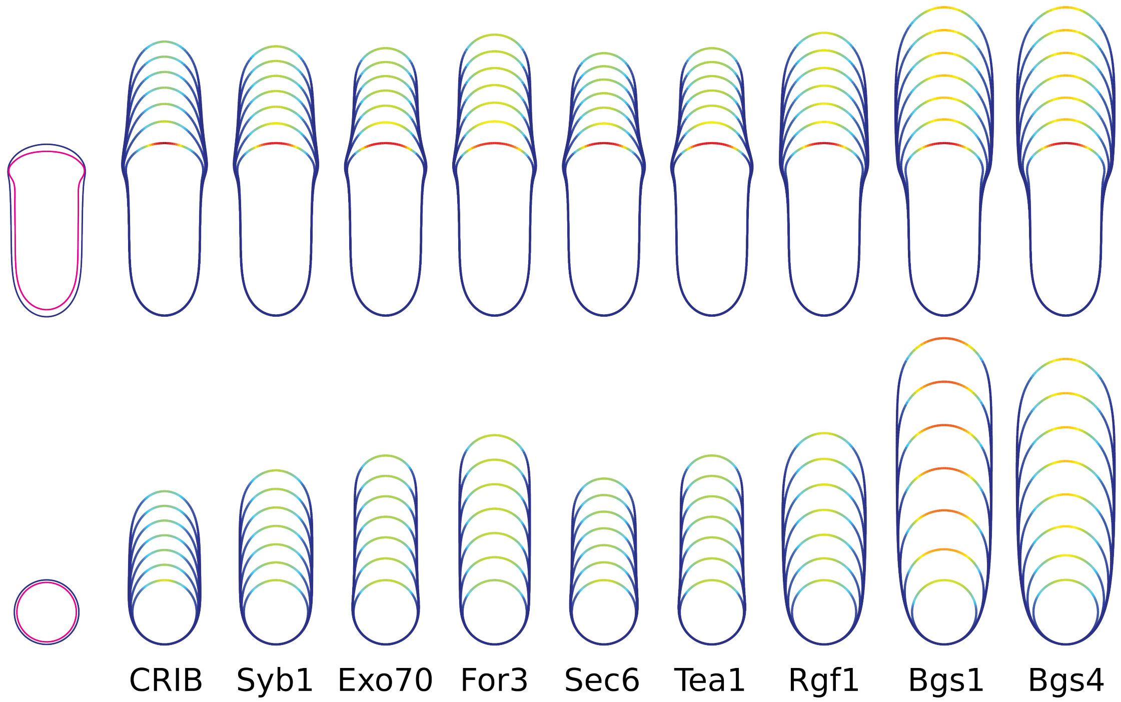

Figure C: Convergence of bending growth model for two initial geometries. The simulations converge to the same steady-state geometry irrespective of the initial cell geometry. The contours on the left represent the relaxed (red) and loaded (blue) geometries used as initial conditions for the simulations.

smaller asymptotic diameter as the main method (the decrease lays between 5% and 10 % depending on the cortical marker) and increases sharply the curvature at the apex giving an aspect pointy which is not physiological. The conclusion of these two controls is that the asymptotic diameter is quite robust whereas the cell end curvature is a litlle less robust and depends on the way the bending term in included in the growth equations.

Simulating cell division - To simulate cell division, an axisymmetrical shell whose profile is an idealized *S. pombe* shape is swelled using the material properties estimated from the simulation of the plasmolysis experiments (see above). An initially straight septum is added at the middle of the swelled cell. The rest length of the septum can be tuned by setting $\lambda_{11} = \lambda_{22} = \lambda^*$ for the septum in equation (42). The size and the curvature Γ_{trans} of the transition between the horizontal septum and the vertical cell wall can also be tuned. The value of the rest length in this zone of transition varies linearly between the value of the swelled cell wall at the insertion of the septum and the value in the septum. The shape of the septum once equilibrated with turgor is obtained by solving for the shell equations listed above.

Two independent parameters have to be estimated for this model: the curvature of the transition Γ_{trans} and the rest length of the septum λ^*. Γ_{trans} was estimated by fit-

ting circles to the transition zone between the septum and the sides of the cell (Figure D, panel b). The average curvature is: $2.27 \pm 0.11 \mu m^{-1}$ ($n = 39$). λ^* was estimated indirectly. Simulations were run for the measured value of Γ_{trans} and for a wide range of λ^*. For each simulation, different measurements of the deformed septum geometry were recorded: D_s the diameter after division, D_0 the diameter before division, H the height of the bulged septum, Γ_{ap} the curvature at the apex. The same parameters were measured on cell images after and before division. First the contour was taken on the brightfield images (the selected points were located at the middle of the black thick line at the contour of the cell). As it was difficult to determine where were the septum extremities after division, the diameter after and before division was calculated by a geometrical method (see Figure D, panel a). The contour was first symmetrized by looking for the best fit by a symmetrical contour. Then the symmetrized contour was oriented vertically (the coordinate x is along the radius, y is along the long axis of the cell). Contour before and after division were superimposed. The daughter cell was divided in two half parts: an old part far from the division plane and a division zone. Daughter cells, whose old part did not superimpose well with the mother cell, were first excluded. The diameter after division was automatically calculated as the maximum of the x coordinates of the divided contour in the division zone. The coordinate of this maximum after division are x_{max} and y_{max} .The diameter before division was chosen as the x coordinate of the contour before division situated at y_{max}.The average ratio between the two diameters was 1.029 ($n = 27$). Then to include more cells in the statistics, a dilatation factor which best superimposed the two non-dividing zones was calculated. This dilatation factor was then used to dilatate the whole contour. The measurements were then repeated. The average ratio only changed slightly (1.034) ($n = 39$). H and Γ_{ap} were estimated on the same contours. The same rest length λ^* was sufficient to predict the different geometrical parameters of the bulging septum (Figure D).

Comparison of the results for the membrane and bending models

Finally, we compared the membrane and bending models to ascertain that our results are robust. Both the asymptotic radius and asymptotic curvature of the cell end are in good agreement between the two models (Table and Figure E).

Table 1: Comparison of the membrane and bending models. The values reported are the percent deviation from the average turgid cell radius of 2.15±0.02 μm ($n = 38$).

Markers	CRIB	Syb1	Exo70	For3	Sec6	Tea1	Rgf1	Bgs1	Bgs4
Membrane model	3%	6%	1%	7%	-14%	-11%	27%	37%	27%
Bending model	-1%	2%	-12%	0%	-11%	-13%	18%	35%	35%

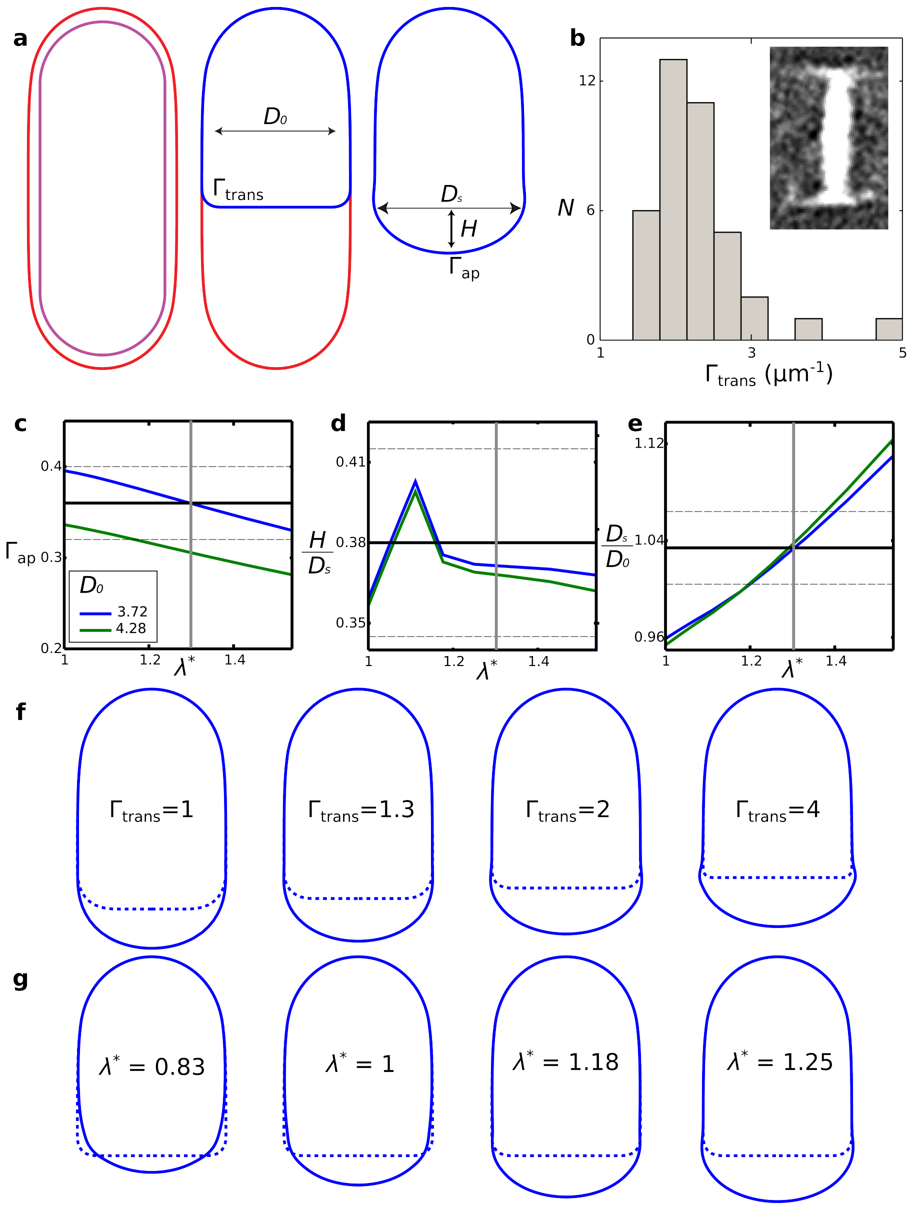

Figure D: Modelling cell division. (**a**) Plasmolysed (magenta) and turgid (red) cell contours. The upper daughter cell (blue) is then showed before cell separation (middle panel) and after separation (right panel). (**b**) Histogram of the measured curvature Γ_{trans} at the transition between septum and cell shaft. (**c**) to (**e**) The apex curvature Γ_{ap}, the height ratio H/D_s, and the diameter ratio D_s/D_0 versus rest length of the septum $\lambda*$ for two different swelling diameter D_0. In the three figures, the black horizontal line corresponds to the average of the observed values ($n = 39$ cells), while the two dashed horizontal lines corresponds to the upper and lower standard deviations for the sample. The vertical line correspond to the value of λ^* which best describes the mechanical behavior of the septum. (**f**) and (**g**) The results of simulations for increasing values of curvature Γ_{trans} and λ^*.

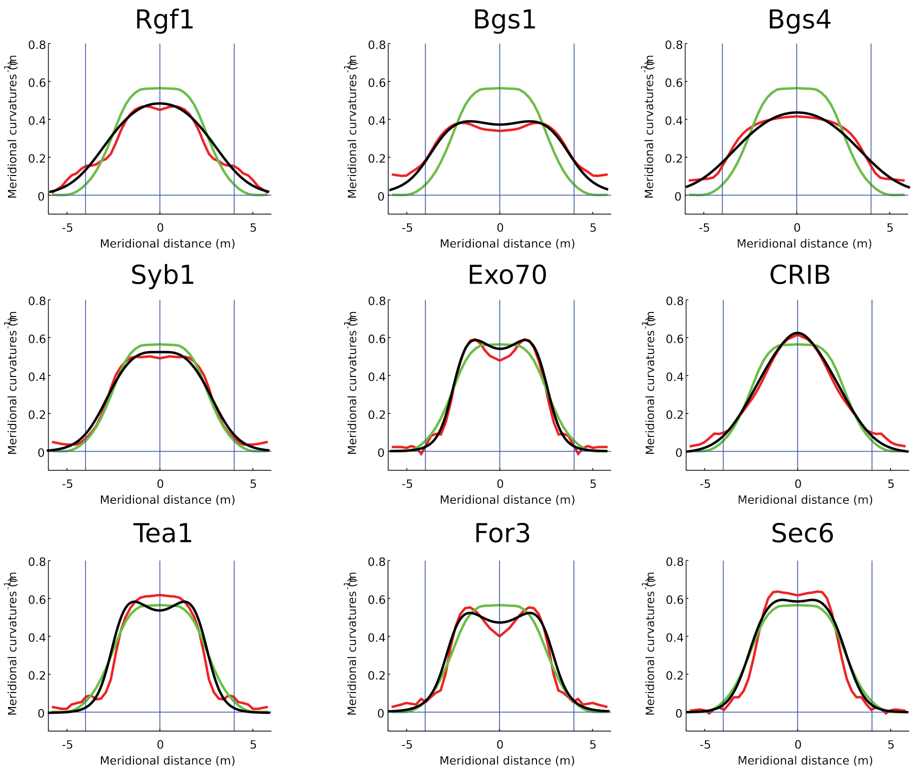

Figure E: Asymptotic meridional curvature of the cell end. The green lines correspond to the measured canonical curvature of the OE. The red lines correspond to the predicted cell end curvature for the membrane model. The black lines correspond of the predicted cell end curvature for the bending model.

SUPPLEMENTARY REFERENCES

1. Kim, D.-U. *et al*. Analysis of a genome-wide set of gene deletions in the fission yeast Schizosaccharomyces pombe. *Nat. Biotechnol.* **28,** 617–623 (2010).
2. Cortés, J. C. G. *et al*. The (1,3)beta-D-glucan synthase subunit Bgs1p is responsible for the fission yeast primary septum formation. *Mol. Microbiol.* **65,** 201–217 (2007).
3. Cortés, J. C. G. *et al*. The novel fission yeast (1,3)beta-D-glucan synthase catalytic subunit Bgs4p is essential during both cytokinesis and polarized growth. *Journal of Cell Science* **118,** 157–174 (2005).
4. García, P., Tajadura, V., García, I. & Sánchez, Y. Rgf1p is a specific Rho1-GEF that coordinates cell polarization with cell wall biogenesis in fission yeast. *Mol. Biol. Cell* **17,** 1620–1631 (2006).
5. Bendezú, F. O., Vincenzetti, V. & Martin, S. G. Fission Yeast Sec3 and Exo70 Are Transported on Actin Cables and Localize the Exocyst Complex to Cell Poles. *PLoS ONE* **7,** e40248 (2012).
6. Bendezu, F. O. & Martin, S. G. Actin cables and the exocyst form two independent morphogenesis pathways in the fission yeast. *Mol. Biol. Cell* **22,** 44–53 (2011).
7. Das, M. *et al*. Oscillatory dynamics of Cdc42 GTPase in the control of polarized growth. *Science* **337,** 239–243 (2012).
8. Smith E.A., Zhang Z., Thomas C.R., Moxham K.E., Middelberg A.P. The mechanical properties of *Saccharomyces cerevisiae*. *Proc Natl Acad Sci U S A*. 97: 9871-9874 (2000).
9. Dumais J., Shaw S.L., Steele C.R., Long S.R. & Ray P.M. An anisotropic-viscoplastic model of plant cell morphogenesis by tip growth. *Int J Dev Biol*. 50:

209222 (2006).

10. Rojas E.R., Hotton S. & Dumais J. Chemically-mediated mechanical expansion of the pollen tube cell wall. *Biophys J*. 101: 1844-1853 (2011).
11. Goriely A., Tabor M. & Tongen A. A morphoelastic model of hyphal tip growth in filamentous organisms. *IUTAM Symposium on Cellular, Molecular and Tissue Mechanics*. 16: 245-255 (2010).
12. Su F.C. & Taber L.A. Torsional boundary layer effects in shells of revolution undergoing large axisymmetric deformation. *Compu Mech*. 10: 23-37 (1992).
13. Kempski M.H., Taber L.A. & Su F. Large elastic deformation of shear deformable shells of revolution: Numerical and experimental results. *J Appl Mech*. 55: 629-534 (1988).
14. Reissner E. On axisymmetrical deformations of thin shells of revolution. *Proc Symp Appl Math*. 3: 27-52 (1950).
